# Plastidial acyl carrier protein Δ9‐desaturase modulates eicosapentaenoic acid biosynthesis and triacylglycerol accumulation in *Phaeodactylum tricornutum*

**DOI:** 10.1111/tpj.15231

**Published:** 2021-05-05

**Authors:** Richard Smith, Juliette Jouhet, Chiara Gandini, Vladimir Nekrasov, Eric Marechal, Johnathan A. Napier, Olga Sayanova

**Affiliations:** ^1^ Department of Plant Sciences Rothamsted Research Harpenden Herts AL5 2JQ UK; ^2^ Laboratoire de Physiologie Cellulaire et Végétale Univ. Grenoble Alpes CNRS IRAE CEA IRIG Grenoble 38000 France; ^3^ Present address: Algenuity Eden Laboratory Broadmead Road Stewartby MK43 9ND UK; ^4^ Present address: Open Bioeconomy Laboratory Department of Chemical Engineering and Biotechnology University of Cambridge Cambridge CB3 0AS UK

**Keywords:** *Phaeodactylum tricornutum*, Δ9‐desaturase, eicosapentaenoic acid, lipids, omega‐3 PUFA biosynthesis

## Abstract

The unicellular marine diatom *Phaeodactylum tricornutum* accumulates up to 35% eicosapentaenoic acid (EPA, 20:5n3) and has been used as a model organism to study long chain polyunsaturated fatty acids (LC‐PUFA) biosynthesis due to an excellent annotated genome sequence and established transformation system. In *P. tricornutum*, the majority of EPA accumulates in polar lipids, particularly in galactolipids such as mono‐ and di‐galactosyldiacylglycerol. LC‐PUFA biosynthesis is considered to start from oleic acid (18:1n9). EPA can be synthesized via a series of desaturation and elongation steps occurring at the endoplasmic reticulum and newly synthesized EPA is then imported into the plastids for incorporation into galactolipids via an unknown route. The basis for the flux of EPA is fundamental to understanding LC‐PUFA biosynthesis in diatoms. We used *P. tricornutum* to study acyl modifying activities, upstream of 18:1n9, on subsequent LC‐PUFA biosynthesis. We identified the gene coding for the plastidial acyl carrier protein Δ9‐desaturase, a key enzyme in fatty acid modification and analyzed the impact of overexpression and knock out of this gene on glycerolipid metabolism. This revealed a previously unknown role of this soluble desaturase in EPA synthesis and production of triacylglycerol. This study provides further insight into the distinctive nature of lipid metabolism in the marine diatom *P. tricornutum* and suggests additional approaches for tailoring oil composition in microalgae.

## Significance Statement

In the marine diatom *Phaeodactylum tricornutum,* the main chloroplast lipids are enriched in eicosapentaenoic acid (EPA) despite its synthesis on the endoplasmic reticulum. We show that committed EPA biosynthesis commences with the action of a plastidial acyl carrier protein Δ9‐ desaturase, using palmitic acid (16:0) as substrate. Targeted mutagenesis of this gene leads to an EPA increase and decreased triacylglycerol, providing evidence that this plastidial enzyme acts as a gating enzyme for extraplastidial EPA and triacylglycerol production.

## INTRODUCTION

In recent years microalgae have attracted considerable attention as an alternative and sustainable platform to produce high value lipids. However, biotechnological improvement of algal strains requires further advances in our understanding of algal physiology and metabolic pathways, coupled with the refinement of molecular toolkits. Heterokonts represent a major group of microalgae containing >16 000 species and are major constituents of the collective biomass known as phytoplankton. Diatoms are thought to have derived from a secondary endosymbiotic event when a red alga was engulfed by a eukaryotic host cell. This led to the formation of secondary plastids surrounded by four membranes, corresponding to the exosymbiont endomembrane, the plasma membrane of the engulfed alga, and the two membranes of the primary plastids (Prihoda *et al.,* 2012). It was shown that in heterokont species the outermost secondary chloroplast limiting membrane forms a continuum with the outer membrane of the endoplasmic reticulum (ER) (Flori *et al.,* 2016). The chloroplast lipid profiles are characterized by the presence of four major lipid classes such as monogalactosyldiacylglycerol (MGDG), digalactosyldiacylglycerol (DGDG), sulfoquinovosyldiacylglycerol (SQDG) and phosphatidylglycerol (PG).

Recently, comprehensive studies of the glycerolipid content of two *Phaeodactylum tricornutum* ecotypes, Pt1 and Pt4, have demonstrated that the glycerolipid profiles are dominated by MGDG, SQDG, DGDG and phosphatidylcholine (PC) (approximately 75% of the total glycerolipid content) while non‐polar lipids, diacylglycerols (DAG) and triacylglycerols (TAG), represent approximately 3% of the total glycerolipids (Abida *et al.,* 2015; Popko *et al.,* 2016). In many heterokonts species, thylakoid lipids contain the long chain polyunsaturate, eicosapentaenoic acid (EPA, 20:5n3) (Abida *et al.,* 2015; Liang *et al.,* 2014). EPA synthesis occurs in the ER (Guschina and Harwood, 2006), implying the existence of unknown pathway(s) for its trafficking from the ER into the chloroplast. The current understanding of lipid metabolism and fatty acid (FA) synthesis in algae is based on genomic analysis and metabolic models derived from higher plants. However, as higher plants neither contain EPA nor display the hallmarks of secondary endosymbionts, such parallels have their limitations. In terms of commonality, *de novo* FA synthesis in diatoms occurs in plastids and leads to formation of a C16‐acyl carrier protein (ACP) by the FA synthase of type II (Guschina and Harwood, 2006). In plants, the C16‐ACP is then: (i) retained in the plastids where it can be esterified by chloroplastic acyltransferase to glycerol‐3‐phosphate (G3P) for conversion into organellar membrane lipids (the so‐called “prokaryotic” pathway); (ii) hydrolyzed from ACP by specific fatty acyl‐ACP thioesterases (FATs) that release free FAs (FFAs) in the inner envelope of the chloroplast; or (iii) further elongated to C18‐ACP. Alternatively, 16:0‐ and 18:0‐ACPs can undergo further desaturation by the soluble ACP desaturases of the chloroplast stroma (Shanklin *et al.,* 2009) and subsequently released by FATs. So far, no FATs similar to that of plants and bacteria have been identified in diatoms (Gong *et al.,* 2011). The formation of C16‐18 FFA is considered the final step of *de novo* plastidial FA biosynthesis followed by the export of these FAs to the cytosol after being esterified to coenzyme A (CoA) by a long‐chain acyl‐CoA synthase located in the outer envelope of the plastid to form acyl‐CoAs (Li‐Beisson *et al.,* 2019; Schnurr *et al.,* 2002). These neosynthesized FAs may be further elongated or desaturated in the ER and used for glycerolipid synthesis (the so‐called “eukaryotic pathway”). In plants, desaturation of 18:0 can be catalyzed either by the soluble stearoyl‐ACP Δ9‐desaturase (SAD) of the chloroplast stroma (Shanklin *et al.,* 2009) or by an extra‐plastidic ER‐bound acyl‐CoA Δ9‐desaturase (ADS) (Fukuchi‐Mizutani *et al.,* 1998), both generating 18:1n9, although SAD is the dominant pathway. As only small amounts of C18‐FAs could be detected in diatom chloroplast lipids, there is no direct evidence of 18:0‐ACP synthesis in the plastids of diatoms, underlining another difference from plant FA biosynthesis. LC‐PUFA biosynthesis is considered to start with desaturation of 18:1n9 by a Δ12‐desaturase resulting in the production of linoleic acid (LA; 18:2n6), which is subsequently converted into α‐linolenic acid (ALA; 18:3n3) by the action of Δ15‐desaturase. Both LA and ALA are then converted into LC‐PUFAs by the sequential desaturation/elongation reactions of that pathway.

The unicellular marine diatom *P. tricornutum* accumulates up to 35% of EPA and has been used as a model to study LC‐PUFA biosynthesis due in part to a well‐annotated genome sequence (Bowler *et al.,* 2008) and established transformation systems (Apt *et al.,* 1996). In *P. tricornutum*, the majority of EPA accumulates in polar lipids, particularly in galactolipids such as MGDG and DGDG (Abida *et al.,* 2015; Arao *et al.,* 1987; Yongmanitchai and Ward, 1993). Pulse chase experiments in *P. tricornutum* revealed that EPA can apparently be synthesized by a number of different routes with the predominant pathway proceeding via Δ6‐desaturation of LA and ALA and utilizing intermediates of both n6 and n3 pathways (Arao *et al.,* 1994). Two main classes of FA desaturases involved in LC‐PUFA biosynthesis have been previously identified in *P. tricornutum* and functionally characterized in yeast. First, soluble enzymes, exemplified by PtFAD6, adding a double bond to an acyl‐ACP substrate and secondly transmembrane enzymes (such as PtFAD2, PtD6 and PtD5), adding a double bond on acyl‐glycerolipid substrates (Domergue *et al.,* 2003; Domergue *et al.,* 2002). Based on these observations, it was suggested that both Δ6‐ and Δ5‐desaturation and Δ6‐elongation take place in the ER and newly synthesized EPA is then imported into the plastids for incorporation into galactolipids via an unknown route called the “omega pathway” (Petroutsos *et al.,* 2014). The understanding of EPA channeling is fundamental to manipulating LC‐PUFA biosynthesis in diatoms.

To date, the mechanism of FA biosynthesis and export from the plastid in diatoms is still unknown. As the 16:0 and 16:1n7 are the main FA synthesized in *P. tricornutum* chloroplast (Abida *et al.,* 2015) it is possible that the *P. tricornutum* acyl‐ACP desaturase will utilize only 16:0‐ACP substrate, acting specifically as a palmitoyl‐ACP Δ9‐desaturase (PAD) rather than SAD. As 18:1n9 is considered a precursor of LC‐PUFA synthesis in the ER and cannot be derived from 16:1n7, acyl‐ACP ∆9‐desaturase therefore represents an interesting target to modulate the accumulation of 18:0 and all downstream metabolites, including EPA. We overexpressed or disrupted the gene coding for the acyl‐ACP ∆9‐desaturase and analyzed the impact on glycerolipid metabolism, revealing an unexpected effect of this soluble plastidial desaturase on EPA synthesis in the ER and production of TAG. Our results indicate that the omega pathway relies on channeling processes occurring very early in plastidial FA desaturation and that this process can be considered as a rate‐limiting step in TAG synthesis.

## RESULTS

### Generation of transgenic *Phaeodactylum tricornutum* overexpressing an acyl‐ACP Δ9‐desaturase

Based on bioinformatic analysis only one ortholog of the Arabidopsis soluble SAD gene, Phat3_J9316, can be found in the *P. tricornutum* genome (Bowler *et al.,* 2008). It contains predicted N‐terminal bipartite targeting sequences with the conserved amino acid sequence motif “ASAFAP” surrounding the signal peptide cleavage site in diatoms (Gruber, 2015) (Figure S1a), consistent with a stromal location.

The native Phat3_J9316 acyl‐ACP Δ9‐desaturase sequence was chemically synthesized (Genscript, Piscataway, NJ, USA) and cloned into pPhos2 vector (Hamilton *et al.,* 2014) behind the EF2 promoter (Seo *et al.,* 2015). The resulted construct, pPhos2‐PAD (Figure S1b) was used to transform *P. tricornutum* via biolistic microparticle bombardment. In total, 38 zeocin‐resistant colonies were screened by polymerase chain reaction (PCR) for the presence of the transgene, of which 20 were positive. The FA analysis of all PCR‐positive transformants revealed that 14 transgenic cell lines had elevated levels of 16:1n7 compared with that of wild type (WT) (Figure S2a). Analysis of transcript abundance of two representative lines (PtPAD#16 and PtPAD#32) with a clear chemotype of elevated 16:1n7, confirmed expression of the codon optimized gene and no change in endogenous gene expression (Figure S2b). Overexpression (OE) of the acyl‐ACP Δ9‐desaturase gene does not significantly change the total FA accumulation relative to WT, with an average total FA contents per cell of 3.2 nmol FA/million cells when harvested after 3 days of cultivation (Figure S2c).

### OE of acyl‐ACP Δ9‐desaturase in transgenic *Phaeodactylum tricornutum* results in reduced levels of EPA and alteration in lipid profiles

**Figure 1:**
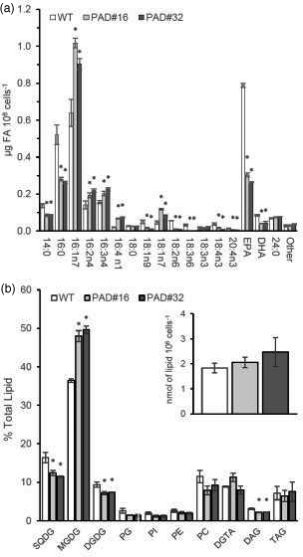
Analysis of fatty acids (FA) and glycerolipids in transgenic *Phaeodactylum tricornutum* overexpressing acyl‐ACP Δ9‐desaturase. (a) Quantitative analysis of FA composition in total lipids (µg FA 10^6^ cells^−1^) in wild‐type (WT) and transgenic palmitoyl‐ACP Δ9‐desaturase (PAD) lines during the E phase. DHA, docosahexaenoic acid; EPA, eicosapentaenoic acid. (b) Glycerolipid classes comparison from WT and transgenic PAD lines during the E phase of growth (3 days). Insert: quantitative analysis of total glycerolipids (nmol of lipid 10^6^ cells^−1^). Values expressed are relative percentage of total lipids. Growth stage was determined by analyzing cell density. Values presented are the average of three biological replicates, error bars represent SE. Asterisks indicate significant difference (*P* < 0.05, LSD) relative to WT. DAG, diacylglycerols; DGDG, digalactosyldiacylglycerol; DGTA, diacylglycerylhydroxymethyltrimethyl‐β‐alanine; MGDG, monogalactosyldiacylglycerol; PC, phosphatidylcholine; PE, phosphatidylethanolamine; PG, phosphatidylglycerol; PI, phosphatidylinositol; SQDG, sulfoquinovosyldiacylglycerol; TAG, triacylglycerols.

The FA profiles of PAD#16 and PAD#32 were further analyzed during the exponential (E) and stationary (S) phases of growth (Figure 1a; Figure S3a). In accordance with previous observations (Hamilton *et al.,* 2014), the major FAs in *P. tricornutum* Pt4 WT cells are palmitic acid (16:0), palmitoleic acid (16:1n7) and EPA. Transgenic lines overexpressing the acyl‐ACP Δ9‐desaturase accumulated significantly higher levels of 16:1n7 compared with that of WT at both growth stages. In the E phase the relative concentration of 16:1n7 is increased from 0.6 µg FA 10^6^ cells ml^−1^ in the WT to 0.9–1.0 µg FA 10^6^ cells ml^−1^ in the OE lines (1.5‐fold, mean of both strains, *P* < 0.05, least significant difference [LSD]), (Figure 1a) and remains elevated in both lines relative to the WT (1.4‐fold, *P* < 0.05, LSD) at S stage (Figure S3a). In addition, the products of further desaturation of 16:1n7, following the route proposed by Domergue *et al.* (2003), 16:1n7 → 16:2n4 → 16:3n4 → 16:4n1, and elongation to 18:1n7 also increased significantly (*P* < 0.05, LSD) in E phase and remained elevated in S phase compared with the WT. An increase in 16:1n7 is correlated with a reduction in the substrate 16:0 (0.52‐fold in the E phase and 0.65‐fold in the S phase, *P* < 0.05, LSD).

Acyl‐ACP Δ9‐desaturase OE resulted in a significant decline of the FA intermediates involved in the proposed predominant route of LC‐PUFA biosynthesis based on radiolabeling experiments (Arao *et al.,* 1994): 18:1n9 → 18:2n6 → 18:3n6 → 18:4n3. This was followed by the significant (approximately 2‐fold, *P* < 0.05, LSD) reduction of the final products of the pathway. Thus, levels of EPA were reduced from 0.79 µg FA 10^6^ cells ml^−1^ in the WT to an average of 0.36 µg FA 10^6^ cells ml^−1^ in E growth phase (Figure 1a) and from 0.5 to 0.25 to µg FA 10^6^ cells ml^−1^ in S stage (Figure S3a). Docosahexaenoic acid (DHA) levels changed from 0.08 to 0.04 µg FA 10^6^ cells ml^−1^ in E growth and 0.04–0.02 µg FA 10^6^ cells ml^−1^ in S growth stages. Together these data demonstrate an important role that that acyl‐ACP Δ9‐desaturase plays in channeling substrates at the very earliest stage of PUFA biosynthesis.

To determine if overexpression of the Δ9‐desaturase gene could alter FA composition and abundance of specific lipid classes, a comprehensive glycerolipidome analysis was carried out. Total lipids were extracted from cells in both the E and S phases. The detection and positional distribution of lipid species were characterized using direct infusion tandem mass spectrometry (MS/MS) with preferential loss analysis after separation by 1D and 2D TLC (Abida *et al.,* 2015). Subsequently, lipid classes and species were quantified from total lipid extracts using high‐performance liquid chromatography‐MS/MS.

The glycerolipid profile of WT *P. tricornutum* Pt4 cells in E phase were largely made up of plastidial membrane lipids, MGDG (36.4%), SQDG (16.4%) and DGDG (9%) and extraplastidial lipid classes such as PC (10.7%), betaine lipid (BL) diacylglycerylhydroxymethyltrimethyl‐β‐alanine (DGTA, 8.9%) and TAG (7.4%) (Figure 1b). In the WT cells grown to S phase, plastidial membrane lipids are degraded, resulting in a reduction in each class (MGDG, 0.39‐fold; DGDG, 0.34‐fold; SQDG 0.53‐fold; PG 0.06‐fold; relative to exponential phase) and TAG is increased 7‐fold (Figure S3b). The glycerolipid profile of Pt4 contrasts with a recent analysis of Popko *et al.* (2016), where major BL class was characterized by MS spectra as diacylglyceroltrimethyhomoserine (DGTS). Three main BLs have been identified in microalgae, DGTA, diacylglyceryl carboxyhydroxymethylcholine and DGTS. DGTA is a structural isomer of DGTS and the two molecules are difficult to differentiate by MS analysis alone. In this study, the presence of DGTA was confirmed by migration on 2D‐TLC and MS/MS fragmentation (Figure S4). The presence and concentration of DGTA is similar to that reported in another *P. tricornutum* ecotype, Pt1 (Abida *et al.,* 2015). We also confirmed the presence of EPA‐acyl‐SQDG, as reported previously; however; this could not be quantified accurately by liquid chromatography‐MS/MS (Abida *et al.,* 2015). The level of TAG in exponential phase of Pt4 was substantially higher (7.4%) than that reported in Pt1 (1–3%, (Abida *et al.,* 2015); however, it is important to note that the level of TAG is highly depended on growth conditions. Comparing the relative amounts of glycerolipids in acyl‐ACP Δ9‐desaturase OE, a significant increase of MGDG was observed in the E stage of cell growth (1.34‐fold, *P* < 0.05 LSD), correlated with decrease in SQDG (0.73‐fold), DGDG (0.78‐fold) and DAG (0.71‐fold) relative to WT (*P* < 0.05 LSD) (Figure 1b). There are no significant differences in proportions of lipid classes between WT and OE lines in the S phase (Figure S3b).

The *sn*‐position of FAs on each glycerolipid was determined by preferential loss using MS and MS/MS analysis as described in Jouhet *et al.* (2017). In agreement with previous observations, EPA is overrepresented in membrane lipids (Abida *et al.,* 2015; Arao *et al.,* 1987; Yongmanitchai and Ward, 1993). Considering chloroplast lipids, the major forms contain a C16 FA at the *sn*‐2 position, suggesting that the prokaryotic pathway for assembling the glycerolipids is very active in diatoms. The most abundant species in MGDG is 36:8, comprising of 20:5 and 16:3 (21%), whereas in DGDG dominant species are 20:5/16:2 (25.5%) and 20:5/16:1 (17.8%), and in PG, the major form is 20:5/16:1 (51%) (Table S1). All these forms have a “prokaryotic” signature, a 20:5 at the *sn*‐1 position and a C16 at the *sn*‐2 position, supporting import of EPA into chloroplast from the ER, followed by plastidial acylation.

The overexpression of acyl‐ACP Δ9‐desaturase resulted in overall decline in all 20:5‐containing species of chloroplast lipids (MGDG, DGDG, SQDG and PG) followed by an increase in C16‐unsaturated containing species at both E and S stages of growth (Tables S1–S4). We observed major reduction in the proportions of 20:5/16:3 form in MGDG (approximately 4‐fold), 20:5/16:0 form in DGDG (approximately 7‐fold) and SQDG (4.8–6.8‐fold) and 20‐5/24:0 form in SQDG (12.6‐fold) compared with WT. Extraplastidial polar lipids, PE, PC and DGTA, contain C16 FA together with C18, C20 and C22 FAs at *sn*‐2 position. This suggests (i) an unbiased FA species incorporation by microsomal acyl‐CoA:lysophosphatidic acid acyltransferase (LPAAT), or (ii) the presence of acyl‐CoA:lysophosphatidylcholine acyltransferase that may synthesize the precursor backbones involved in ER membrane glycerolipid assembly. Noteworthy, 20:5 is present at *sn*‐2 position only in the eukaryotic 20/20:5 form and 22:6 is found only in 20:5/22:6 form (Tables S1–S4). In PE, *sn*‐1 position was mainly occupied by 20:5 acyl group. In the OEs, the greatest reduction was observed in 20:5/C18 and 20:5/LC‐PUFA forms of PC, DGTA and PE with major decline of 20:5/20:5 form in PC and DGTA (from approximately 8.4‐ to 5‐fold and 6.3‐ to 4.3‐fold respectively) and 20:5/22:6 in PC (9.9‐fold). The major alteration observed was an increase in 16:1/16:1 and 16:1/16:2 forms in PC (5.7–8.0 and 8.0–11.4‐fold respectively, pmol 10^6^ cell^−1^) and DGTA (3.7–4.3‐ and 5.8–7.5‐fold respectively, pmol 10^6^ cell^−1^). More surprising was an increase in 20:5/16:1 and 20:5/16:2 forms in PE (5.0–6.0‐ and 6.0–7.4‐fold respectively, pmol 10^6^ cell^−1^) correlated with a decrease of these two major backbones in DGDG (approximately 2–3‐fold), suggesting that 20:5/16:1 and 20:5/16:2 species could be potentially exported as DAG.

Considering non‐polar lipids, the DAG pool is represented by four molecular species, comprising mainly of 16:1, 16:0, 14:0 and 20:5 acyl groups, with 14:0 and 20:5 being found only at the *sn*‐1 position. The DAG acyl composition (Tables S1–S4) partially reflects that found in the main membrane lipids and we cannot rule out the recycling of the diacylglyceride backbone from membrane lipids. The major decline (approximately 6‐fold) has been observed in the 20:5/16:0 form found in most polar lipids followed by an increase in 16:1/16:1.

Three DAG molecular species, 14:0/16:1, 16:1/16:1 and 20:5/16:0, are potential precursors of the TAG pool with *sn*‐3 position occupied by 14:0 or C16 FA. In acyl‐ACP Δ9‐desaturase OE, TAG 20:5‐containing species also declined followed by increase in 16:1 (Tables S1–S4). Similar FA backbone as in TAG, 20:5/16:0 with 20:5 FA at the *sn*‐1 position was also reduced in direct precursor DAG and in PC, DGDG and PG. The most significant alterations were observed in the increased proportions of 16:1/16:1‐ and decrease in 20:5/16:0‐containing TAG species, suggesting that all these lipids may act as substrates for TAG biosynthesis.

### Generation of transgenic *Phaeodactylum tricornutum* with acyl ACP Δ9‐desaturase knock out

**Figure 2:**
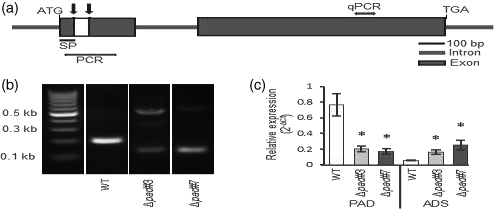
Generation of acyl‐ACP Δ9‐desaturase knockout (KO) strains by CRISPR/Cas9. (a) Schematic structure (important features) of the CRISPR/Cas9 target locus within the Δ9‐ desaturase gene. Positions of guide RNA complementary binding sites in the first exon are indicated with black arrows. Approximate region of nucleotide deletion is shown in white. Gray arrows indicate the site of amplification for polymerase chain reaction (PCR) genotyping and quantitative (q)PCR of cDNA. SP, signal peptide. (b) PCR screen of native acyl‐ACP Δ9‐desaturase gene in KO lines (see Figure S5 for position of primers and expected deletion). Lower MW band shift indicates bi‐allelic deletion. WT, wild type. (c) Transcript abundance of acyl‐ACP Δ9‐desaturase (Phatr3_J9316) and putative Δ9 acyl‐CoA desaturase (ADS) (Phatr3_J28797) genes. Expression is reported relative to the reference gene Aureochrome (Phatr3_J8113). Values presented are the average of three biological replicates, error bars represent SE. Asterisks indicate significant difference relative to WT (*P* < 0.05, LSD). MW, molecular weight; PAD, palmitoyl‐ACP Δ9‐desaturase.

To test the hypothesis that plastidial Δ9‐desaturation of 16:0 acts as a bottleneck for 16:0 export and further elongation and desaturation to EPA, the native Phat3_J9316 gene was targeted for disruption using CRISPR/Cas9 and two sgRNA guides to ensure gene disruption (Figure 2a; Figure S5a). Cells were transformed using the previously described method (Hamilton *et al.,* 2014) with the transformation efficiency approximately 1.6 colonies μg^−1^ plasmid. Among 33 colonies which were screened by PCR, two (∆*pad*#3 and ∆*pad*#7) had a lower molecular weight band, indicating deletion of sequences within the target gene (Figure 2b). The sgRNAs were designed to generate a 57‐nt deletion within the first exon of the gene (Figure S5a). Cloning and subsequent sequencing of the PCR products revealed mosaicism within each knock‐out (KO) lines (Figure S5b–e), including the insertion of a 371‐bp fragment derived from the transformation vector plasmid backbone. The presence of bands >500 bp on the agarose gel in both lines indicates that larger insertions may also be present in the cell population. The relative abundance of each deletion indicates that the deletion happened in multiple events after initial cell division. There was no detection of unaltered DNA sequence between the gRNAs in either ∆*pad*#3 and ∆*pad*#7 lines, confirming a deletion in both these alleles. Consequently, the lines were analyzed without further isolation of sub‐clones. Both KO lines had significantly reduced levels of acyl‐ACP Δ9‐desaturase mRNA, most likely indicating increased post‐transcriptional turnover of the mutated mRNA (Figure 2c).

### FA and glycerolipid analysis of transgenic *Phaeodactylum tricornutum* with disruption of endogenous acyl‐ACP Δ9‐desaturase reveals increases in EPA and major alterations in TAG

**Figure 3:**
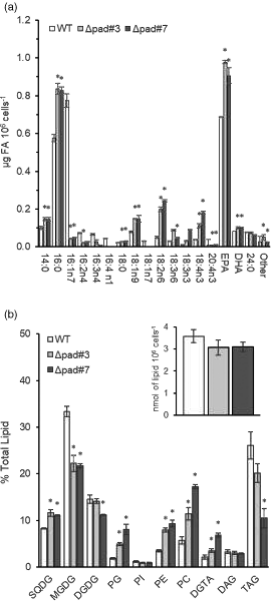
Fatty acid (FA) and glycerolipid classes: comparison in the wild‐type (WT) and knockout strains during the exponential stage of growth phase (3 days). (a) FA content. Values expressed in µg FA 10^6^ cells^−1^. DHA, docosahexaenoic acid; EPA, eicosapentaenoic acid; PAD, palmitoyl‐acyl‐carrier protein Δ9‐desaturase. (b) Glycerolipid classes. Values expressed in percentage of total lipids. Insert: quantitative analysis of total glycerolipids (nmol of lipid 10^6^ cells^−1^). Growth stage was determined by analyzing cell density. Values presented are the average of three biological replicates, error bars represent SE. Asterisks indicate significant difference (*P* < 0.05, LSD) relative to WT. DAG, diacylglycerols; DGDG, digalactosyldiacylglycerol; DGTA, diacylglycerylhydroxymethyltrimethyl‐β‐alanine; MGDG, monogalactosyldiacylglycerol; PC, phosphatidylcholine; PE, phosphatidylethanolamine; PG, phosphatidylglycerol; PI, phosphatidylinositol; SQDG, sulfoquinovosyldiacylglycerol; TAG, triacylglycerols.

The FA profiles of two disrupted/KO lines were analyzed in the exponential and stationary phase of growth. KO of the acyl‐ACP Δ9‐desaturase gene resulted in a significant reduction of 16:1n7 (94% reduction relative to WT) and, consequently, in a smaller pool of products of further desaturation (16:2n4, 16:3n4, 16:4n1) and elongation (18:1n7) in both E and S phases (Figure 3a; Figure S6a). In contrast, the accumulation of 16:0, a substrate for acyl‐ACP Δ9‐desaturase, increased approximately 1.45‐fold followed by a significant increase in the levels of 18:1n9 versus WT, indicating independent desaturation of C18:0 by putative Δ9 acyl‐CoA desaturase. Furthermore, the proportion of many intermediate FAs in extraplastidic PUFA synthesis pathway also significantly increased culminating in an elevated accumulation of EPA (approximately 1.32–1.42‐fold), increasing from 0.69 µg FA 10^6^ cells^−1^ in the WT cultures, to 0.91–0.98 µg FA 10^6^ cells^−1^ in the KO strains. Interestingly, the intermediates most affected by the KO (LA, ALA, 18:4n3, 20:4n3) are those highlighted by Arao et al. to be involved in the dominant route of PUFA biosynthesis in *P. tricornutum* (Arao *et al.,* 1994). The minor increase in proportions of DHA indicates that the pool size of EPA is not a bottleneck for synthesis; rather, as demonstrated by heterologous OtElo5 expression in *P. tricornutum*, accessibility of elongated products in the native strain is restrictive (Hamilton *et al.,* 2014). The residual 16:1n7 found in the KO line profile may be produced in the cytosol by a homolog of Arabidopsis ADS1 (Liu *et al.,* 2020) acting on 16:0‐CoA and imported back into the plastid. We have identified only one candidate Δ9‐oleyl CoA desaturase, PtADS1 (*Phatr3_J29797*) based on the sequence homology. Increased expression of a putative PtADS1 desaturase in KO lines relative to the WT (approximately 2.4‐fold) supports this hypothesis (Figure 2c).

Glycerolipidomic analysis was carried out to determine how disruption of the acyl‐ACP Δ9‐desaturase gene affected the distribution of glycerolipids and FA within each class. The WT profile, sampled in E phase, was largely similar to the previously outlined WT analysis, with the exception of a larger pool of TAG (24.5% versus 7.2% total lipid), which is likely due to slight differences in condition (synchronization) of starting inoculum (Figure 3b). Unlike the OE lines, KO of acyl‐ACP Δ9‐desaturase resulted in a significant shift in the glycerolipid profile. KO lines had a significant reduction in pools of MGDG (0.65–0.67‐fold) and TAG (0.40–0.77‐fold), and significant increases in SQDG (1.34–1.40‐fold), PG (2.64–4.31‐fold), PE (2.30–2.70‐fold), PC (1.99–3.01‐fold) and DGTA (1.65–3.26‐fold). Lipid classes analysis (Tables S6–S10) reveal the major increase in “TAG‐diagnostic” 20:5/16:0 species in main chloroplast lipids, SQDG (4.15–4.85‐fold), MGDG (14.1–15.7‐fold), DGDG (4.17–5.19‐fold) and PG (3.76–7.01‐fold) correlated with a significant decrease in 16:1–containing species and metabolic derivatives (16:2, 16:3 and 16:4) were undetectable. A dramatic increase in C18‐FA was observed in 20:5‐containing lipids in MGDG and SQDG (approximately 49‐fold in 20:5/18:1 and 24–28‐fold in 20:5/18:2 in MGDG; 3–5‐fold increase in 20:5/18:2 in SQDG). The typical eukaryotic backbones, containing C18 FA and 20:4n3 at *sn*‐2 position can come from the recycling of phospholipids such as PC, DGTA and PE similar to galactolipid synthesis in higher plants.

Within the extraplastidial PLs, there was a major increase in unsaturated C18, 20:5 and 22:6 containing forms. The 20:5/20:5 form was slightly elevated in PE (1.5‐fold), PC (1.8‐fold) and DGTA (2‐fold). Interestingly, an increase in 20:5/18:2 forms in PE, PC and DGTA was mirrored by similar changes in MGDG and SQDG, while 20:5/18:1 species concomitantly increased in PC, DGTA, MGDG and SQDG. This may imply that PE, PC and DGTA serve as precursors for galactolipids in *P. tricornutum*. The most significant changes in the DAG pool were increase in 20:5/16:0 form (21.65–22.92‐fold) and disappearance of 16:1/16:1 (non‐detected in KO strains). Alterations in TAG reflected that of membrane lipids and a DAG pool with a noticeable increase in 18:1‐ and 20:5‐containing forms. Significant increase is detected in 20:5/16:0/16:0 (1.68–3.64‐fold) and 20:5/20:5/16:0 (4.31–7.35‐fold) forms.

Concerning changes in FA content, in chloroplast lipids in E phase of cell growth, EPA content increased significantly in SQDG (from 32.7 to 158.5 pmol FA 10^6^ cells^−1^, approximately 5‐fold) and in PG (3–5‐fold) (Table S9) whereas in MGDG it remains unaltered. In extraplastidial lipids the most increase was observed in DAG (approximately 11‐fold) followed by significant increase in PE (1.9–2.2‐fold) and PC (1.5–2.5‐fold). A sharp increase in C18 FAs was detected in SQDG and MGDG, followed by a more moderate uptick in PE, PC and DGTA. 16:1 levels were markedly reduced in all lipids. Interestingly, the most significant decrease in 16:1 was observed in DAG (26–72‐fold), suggesting the DAG pool is produced from neosynthesized FA. A similar pattern of FA accumulation was observed at the S stage. In TAG, the most notable increase of EPA was observed in the stationary phase (approximately 2‐fold), followed by an approximately 4‐fold decrease in 16:1. Collectively, these observations support the hypothesis that the Phat3_J9316 acyl‐ACP Δ9‐desaturase acts as a competitor for 16:0 export and subsequent elongation/desaturation to EPA.

### Heterologous characterization of the acyl‐ACP Δ9‐desaturase in *Synechocystis*

**Figure 4:**
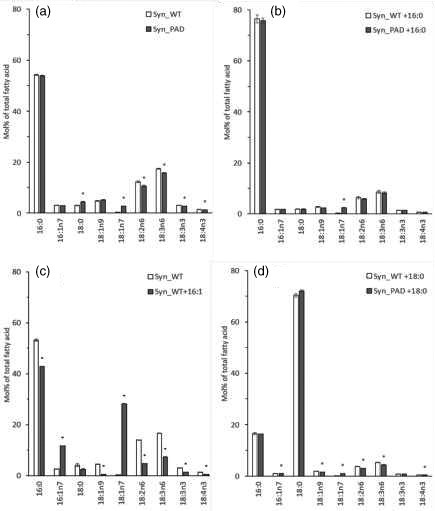
Fatty acid composition (Mol% of total fatty acid) of Syn_WT and Syn_PAD strains after 4 days growth. (a) No fatty acid supplementation. PAD, palmitoyl‐acyl‐carrier protein Δ9‐desaturase; Syn, *Synechocystis*; WT, wild type. (b) Supplementation with 16:0. (c) Syn_WT with and without palmitoleic acid. (d) Supplementation with C18:0. Data values represent the average of three biological replicate, error bars represent SE. Asterisks indicate significant differences relative to WT (*P* < 0.05) in two‐tailed Student’s *t*‐test.

For the functional definition of the Phat3_J9316 acyl‐ACP Δ9‐desaturase activity evaluated above, the cyanobacteria *Synechocystis* PCC6803 strain was used as a heterologous expression system. The Δ9‐desaturase sequence Phat3_J9316 lacking the predicted signal peptide was cloned into the pUR expression vector and expressed in *Synechocystis* to confirm its enzymatic activity (Figure S7). *Synechocystis* has a relatively simple FA profile with 16:0, 16:1n7, 18:0, 18:1n9, 18:2n6, 18:3n6 and 18:3n3 as the major FAs, whereas 18:4n3 and 18:1n7 are found only in trace amounts. The expression of the Phat3_J9316 acyl‐ACP Δ9‐desaturase resulted in decreased levels of nearly all the major FAs except 18:0 and increased presence of 18:1 n7, the expected product of elongation of 16:1n7 (Figure 4a). The same trend was observed when the cultures were exogenously supplied with 16:0 (Figure 4b). To confirm the origin of 18:1n7, WT *Synechocystis* cultures were supplemented with 16:1n7 and grown until E phase before FA analysis (Figure S8b). Exogenously supplied 16:1n7 only slightly increased the content of 16:1n7, but the levels of 18:1n7 were significantly increased, confirming the presence of a very efficient elongation of 16:1n7 to 18:1n7 in *Synechocystis* cells (Figure 4c). Supplementation of 18:0 to WT and PAD expressing *Synechocystis* cultures did not lead to an increase in Δ9 desaturation (Figure 4d), confirming the gene is specific to palmitic acid.

### Altered ultrastructure of thylakoid membranes in transgenic *Phaeodactylum tricornutum* strains

Cross‐sections of chloroplasts of E stage cells were analyzed by transmission electron microscopy to assess the impact of acyl‐ACP Δ9‐desaturase OE or inactivation on the ultrastructure of thylakoid membranes. WT chloroplasts contained several parallel thylakoid lamellae, each consisting of a stack of three membrane bilayers (Figure S8). Both mutants have disturbed thylakoid structure. The OE PAD#12 chloroplasts, containing more MGDG, have a similar number of stacks, but they exhibit a larger luminal space with increased packing of stacks and more extreme curvature of the stacks (red arrow) and less parallelism between membranes is observed. In KO Δpad#3 chloroplasts, containing less MGDG, thylakoids are distorted, less electron dense than that of WT (blue arrow) with expanded inter‐space, reduced stroma space and curvature of the lamella (green arrow), and no obvious pyrenoid is present. Surprisingly, no differences were observed in specific growth rates between OE, KO and WT cells, suggesting that changes in thylakoid structure has no impact on the growth under normal conditions (Figure S9).

## DISCUSSION

Our study confirms that in the marine diatom *P. tricornutum* Pt4 the main FAs synthesized *de novo* in the stroma of chloroplasts are 14:0‐ and 16:0‐ACP (Abida *et al.,* 2015; Popko, 2016). We identified one sequence in *P. tricornutum* genome, Phat3_J9316, containing both a predicted signal peptide and chloroplast transit peptide with a diatom‐specific ASAFAP motif, and orthologous to the Arabidopsis SAD gene. However, multiple lines of evidence support the classification of the Phat3_J9316 desaturase sequence as a PAD as opposed to SAD. First, the absence of 18:0 in *P. tricornutum* plastid lipids suggests that a soluble Δ9‐acyl‐ACP desaturase would act predominantly on 16:0‐ACP, generating 16:1n7‐ACP. Overexpression of Phat3_J9316 leads to an increase in 16:1n7 correlated with reduced levels of 16:0 and 18:1n9, indicating that the enzyme has a strong preference to 16:0 as a substrate. In addition, we provided additional evidence for the substrate specificity of Phat3_J9316 by functional characterization in the heterologous host *Synechocystis*, which showed an increased accumulation of 18:1n7 in Phat3_J9316 overexpressing cells, is because of Δ9‐desaturase activity on 16:0 and the product of elongation of 16:1n7. Although such heterologous assays do not provide direct biochemical evidence of substrate preference, they strongly support this conclusion. In addition, in *P. tricornutum* KO mutants where Phat3_J9316 is disrupted by CRISPR‐Cas9 gene‐editing, there is a significant increase in levels of 16:0 consistent with the loss of PAD activity. Based on these results, we have assigned the function of PAD to Phat3_J9316. Recently, Liu et al. demonstrated that transient expression of Phat3_J9316 in *Nicotiana benthamiana* leaves was accompanied by the accumulation of 16:1Δ9 (Liu *et al.,* 2020). When the Phat3_J9316 sequence was aligned with 18:0‐ACP–specific castor RcSAD1 (Lindqvist *et al.,* 1996) and *Arabidopsis thaliana* AtFAB2 (Troncoso‐Ponce, 2016) and 16:0‐ACP–specific *A. thaliana* AtAAD3, AtAAD2 (Bryant *et al.,* 2016) and cat’s claw Muc‐PAD (Cahoon *et al.,* 1998) Δ9‐desaturases, three variant AA residues (F160, A223 and L156) of the key eight amino acids were found similar to that of 16:0‐ACP specific isoforms, suggesting that this desaturase may have the substrate specificity for 16:0‐ACP (Whittle and Shanklin, 2001). This was confirmed by transient expression of a mutated version (F160L, A223T and L156M) of Phat3_J9316 in *N. benthamiana* leaves resulting in increased levels of 18:1Δ9 without accumulation of 16:1Δ9 (Liu *et al.,* 2020) and providing additional independent evidence for our assignment of Phat3_J9316 as PAD.

**Figure 5:**
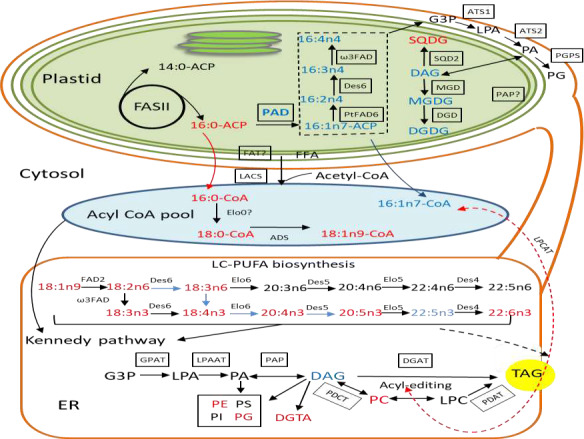
Proposed pathway for EPA biosynthesis and possible EPA import routes from the ER into the chloroplast of *Phaeodactylum tricornutum*. Red text indicates increased pool size of metabolite or activity of enzyme in knockout cells. Blue text indicates reduced pool size or activity of enzyme. *de novo* synthesized 16:0‐ACP enters the prokaryotic pathway where it can be used in the plastid for the production of MGDG, DGDG, SQDG and PG via a canonical prokaryotic pathway or desaturated by PAD to 16:1n7‐ACP and further by soluble ACP desaturases. When exported to the cytosol through the long‐chain acyl‐CoA synthases pathway to enter the eukaryotic pathway, C16‐ACPs are hydrolyzed by unknown FAT and FFA are exported through the plastidial membrane to enter CoA pool. 16:0‐CoA in elongated to 18:0‐CoA with further desaturation by homolog of Arabidopsis ADS1 to produce 18:1Δ9‐CoA, which is at the base of PUFA biosynthesis. Newly produced EPA could be incorporated into acyl‐CoA and imported from ER into the chloroplast for galactolipid synthesis. From the ER, EPA could be imported into the chloroplast as incorporated into PA or DAG. EPA can be converted into TAG by an acyl‐independent PDAT after acyl‐editing or enter the Kennedy pathway and be converted into TAG by DGAT. ACP, acyl carrier protein; ADS1, Δ9‐acyl‐CoA desaturase; ATS1, plastidic glycerol‐3‐phosphate acyltransferase; ATS2, plastidic lysophosphatidic acid transferase; CoA, coenzyme A; DAG, diacylglycerols; DGAT, acyl‐CoA:diacylglycerol acyltransferase; DGDG, digalactosyldiacylglycerol; DGTA, diacylglycerylhydroxymethyltrimethyl‐β‐alanine; ER, endoplasmic reticulum; FAD, fatty acid desaturases; FASII, fatty acid synthase of type II; FAT, fatty acyl‐ACP thioesterases; FFA, free fatty acids; G3P, glycerol‐3‐phosphate; GPAT, glycerol‐3‐phosphate acyltransferase; LACS, long chain acyl‐coenzyme A synthetase; LC‐PUFA, long chain polyunsaturated fatty acids; LPA, lysophosphatidic acid; LPAAT, lysophosphatidic acid acyltransferase; LPC, lysophosphatidylcholine; LPCAT, lysophosphatidylcholine acyltransferase; MGDG, monogalactosyldiacylglycerol; PA, phosphatidic acid; PAD, palmitoyl‐ACP Δ9‐desaturase; PAP, phosphatidic acid phosphatase; PC, phosphatidylcholine; PDAT, phospholipid:diacylglycerol acyltransferase; PDCT, phosphatidycholine:diacylglycerol cholinephosphotransferase; PE, phosphatidylethanolamine; PG, phosphatidylglycerol; PGPS, phosphatidylglycerol phosphate synthase; PI, phosphatidylinositol; PS, phosphatidylserine; SQDG, sulfoquinovosyldiacylglycerol; TAG, triacylglycerols.

Overexpression of this PAD gene in *P. tricornutum* leads to an overall reduction of the FA intermediates (Figure S10) in the presumed predominant LC‐PUFA biosynthetic pathway starting from 18:1n9 (Arao *et al.,* 1994) and 2‐fold decline in EPA and DHA, indicating that *PAD* plays a key role in channeling processes from the very early stage of PUFA biosynthesis and the desaturation of 16:0 in plastid likely to be a “crossroad” for acyl flux through to 20:5. This is supported by the enhanced accumulation of 20:5 and concomitant reduction in 16:1 in stationary phase TAGs of PAD KO cells (Figure 5). Δ9‐desaturation via PAD also modulates the levels of MGDG, the OE cells having higher MGDG levels while KO cells have lower, although both types of mutants displayed disturbed thylakoid membrane structures.

The inverse relationship between levels of 16:0/16:1 and EPA in OE and KO mutants, and the minor presence of C18 FA within chloroplast lipids, suggests that 16:0 is exported to the cytosol where it is converted into 16:0‐CoA for further elongation and desaturation. This is in agreement with our previous observations that 20:5‐CoA, 16:1‐CoA and 16:0‐CoA are the most abundant acyl‐CoA species in *P. tricornutum* (Hamilton *et al.,* 2014 *)*. Recently, Dolch *et al*. demonstrated that *Nannochloropsis gaditana* saturated FA elongase, Δ0‐ELO1, could elongate palmitic acid (Dolch *et al.,* 2017). *N. gaditana* Δ0‐elo1 mutants exhibited a reduced EPA level and a specific decrease in MGDG. The study provided evidence that some of EPA used for MGDG production is biosynthesized by a channeled process initiated at the elongation step of palmitic acid by Δ0‐ELO1, thus acting as a channeling enzyme for galactolipid production despite residing in the ER (Dolch *et al.,* 2017). We identified two putative saturated ER FA elongases, Phatr3_J16376 (Δ0‐Elo1) and Phatr3_J49867 (Δ0‐Elo02), homologs of *N*. *gaditana* ER palmitic acid elongase Δ0‐ELO1 (Dolch *et al.,* 2017), which could presumably elongate 16:0‐CoA to 18:0‐CoA. This indicates that diatoms developed a different route of biosynthesis from plants, where the canonical conversion of 16:0 to 18:0 is carried out by plastidial a β‐ketoacyl‐ACP synthase II (KASII). Recently, Aslan *et al*., demonstrated that KO of KASII in tobacco resulted in increased accumulation of C16 FAs (Aslan *et al.,* 2015), suggesting that plants do not have the ability to utilize ER‐located elongase for conversion of plastid 16:0 to 18:0. In the *P. tricornutum* genome a KASII ortholog has been annotated but not functionally characterized. Further modification of 18:0‐CoA could be carried out by orthologs of the Arabidopsis ADS1, to produce 18:1Δ9‐CoA. We identified only one putative Δ9‐oleyl desaturase sequence, Phatr_28797, with an N‐terminal sequence consistent with an ER localization. The present results suggest that acyl‐CoA desaturase participates in desaturation at the Δ9 position of 18:0 in the ER. The role of two putative Δ0‐elongases and Δ9‐oleyl desaturase in LC‐PUFA biosynthesis is under investigation.

In higher plants, *de novo* plastidial 16:0‐ and 18:0‐ACPs can enter two distinct routes of lipid synthesis, the “prokaryotic” or “eukaryotic.” The “prokaryotic” or “eukaryotic” structure of plastid lipids is determined by the origin of phosphatidic acid (PA) and DAG. In plants, FAs synthesized in the chloroplast are transferred on to G3P by a plastidial acyl‐G3P acyltransferase (ATS1). Further esterification by the plastid LPAAT (ATS2), leads to the production of PA and DAG with C18 and C16 at position *sn*‐1 and *sn*‐2 respectively. Chloroplast desaturases acting on 16:0 produce a range of C16 unsaturated FAs, found at the *sn*‐2 position of MGDG and DGDG lipids. In the “eukaryotic” pathway, acyl‐ACPs are hydrolyzed by FAT to produce free FAs, which are exported to the ER for the subsequent incorporation into membrane glycerolipids with C18 at *sn*‐2 position before re‐import to the chloroplast. The presence of a eukaryotic pathway involving recycling of DAG backbone coming from phospholipid for galactolipid synthesis, as described in higher plants, has not been demonstrated in heterokonts. In *P. tricornutum*, EPA found in chloroplast lipids will have been synthesized in the ER by ER‐located desaturases and elongases (Domergue *et al.,* 2002). Thus, EPA and other LC‐PUFAs must also be re‐imported to secondary plastids for incorporation into the plastidial glycolipids. However, the nature of precursors that are transported over the chloroplast‐limiting membranes remains unknown.

In all analyzed chloroplast lipids of WT and mutant cells, EPA is exclusively found in the *sn*‐1 position whereas C16 occupy mainly the *sn*‐2 position, confirming the observation that the prokaryotic pathway for the synthesis of the glycerolipids is dominant in heterokonts (Abida *et al.,* 2015; Dolch *et al.,* 2017; Simionato *et al.,* 2013). This also suggests that the plastid LPAAT has a very high selectivity for C16 FAs as the acyl donors to generate *sn*‐2‐C16‐PA giving rise to lipids with the prokaryotic signature. ER membrane glycerolipids of WT and both mutants, such as PC, DGTA and PE mostly have C18, C20 and C22 FA together with C16 species esterified at the *sn*‐2 position indicating that either microsomal LPAAT has no selectivity for FA species or acyl‐CoA:lysophospholipid acyltransferase may be involved in ER membrane glycerolipid assembly.

In the different *P. tricornutum* cells used in this study, TAGs are always enriched in C16 FAs at their *sn*‐2 position implying that the TAG synthesis may be similar to that described in *Chlamydomonas reinhardtii* where a chlorophyte‐specific acyltransferase, CrLPAAT2, localized to the ER, prefers 16:0‐CoA as the substrate for synthesis of glycerolipid intermediates for TAG assembly and/or transfer to the plastid (Kim *et al.,* 2016). As C18 and C20 are hardly detected at the *sn*‐2 position in thylakoid lipids in WT and OE cells, similar selectivity may operate in *P. tricornutum*, implying that ER‐synthesized precursors (C16 bound to *sn*‐2‐PA) have diagnostic C16 acyl chains esterified at this position. A >20‐fold increase in specific signature 20:5/16:0 form in DAG of KO cells followed by similar increase in this form in thylakoid lipids supports this hypothesis. Interestingly, MGDG and SQDG in KO cells contain increased levels of C18 bound to *sn*‐2 molecular species concomitant with an increase in these forms (16:1/18:1; 20:5/18:1, 20:5/18:2) in PE, PC and DGTA suggesting the potential existence of a eukaryotic pathway with the recycling of diacyl precursors coming from phospholipid for galactolipid synthesis, as described in higher plants and *C. reinhardtii* (Fan *et al.,* 2011). Alternatively, ER located PA and DAG may serve as precursors of galactolipid and sulfolipid synthesis.

An increase of 16:1 and concomitant reduction in EPA levels in TAG in OE cells may indicate that *de novo* FA synthesis contributes to TAG biosynthesis via the Kennedy pathway involving a diacylglycerol acyltransferase and *de novo* DAG and acyl‐CoA synthesis. This may be supported by the observed decrease in 16:1 and increase in EPA levels in KO cells. Despite the significant changes to the FA profile, OE or KO of the *PAD* gene does not significantly change the total FA accumulation relative to WT, indicating no change in *de novo* FA synthesis. In addition, the DAG acyl composition does not fully reflect the composition of the main membrane lipids in support of a *de novo* synthesis and, in contrast to plants, does not indicate any substantial recycling of DAG moieties deriving from membrane glycerolipids (Bates and Browse, 2012). ER‐synthesized EPA may be also imported into chloroplast and incorporated into glycolipids by the suggested “omega pathway.” Newly formed EPA may be released from a phospholipid into the cytosolic acyl‐CoA pool and then transported into the chloroplasts to be attached to MDGD at the *sn*‐1 position by the acyltransferase, ATS1 (Petroutsos *et al.,* 2014).

### Conclusions

The role of EPA in chloroplast lipids remains unknown. We identified and characterized the gene coding for the plastidial ACP Δ9‐desaturase, a key enzyme in FA modification. The analyzed impact of OE and KO of this gene on glycerolipid metabolism revealed a previously unknown role for this soluble desaturase in EPA synthesis and the production of TAG.

We propose that the PAD gene acts as a key factor in determining EPA levels to maintain phenotypic plasticity. In particular, the modulation of both MGDG and TAG levels through the presence/absence of 16:1n7 represents a simple “tag” by which different lipids can be sorted for further modification. This study provides further insight into the distinctive nature of lipid metabolism in the marine diatom *P. tricornutum* and suggests an additional approach for tailoring oil composition in microalgae.

## EXPERIMENTAL PROCEDURES

### Strain and culture conditions

*Phaeodactylum tricornutum* Pt4 strain (*UTEX 646*) was grown in artificial sea water (Instant Ocean, Spectrum Brands, Blacksburg, VA, USA) supplemented with F/2 nutrients. Cultures were grown at 20°C under constant illumination (100 µmol m^−2^ sec^−1^, 4000 K White and 660 nm LED lighting) and agitated continuously at 70 rpm. Growth was monitored by OD_750nm_ calibrated to cell density measured by an automated cell counter (Cellometer T4, Nexcelom, Lawrence, MA, USA). Lines were maintained on F/4 agar plates grown at 20°C under 50 µmol m^−2^ sec^−1^ (3500 K fluorescent tubes).

### Construction of acyl‐ACP Δ9‐desaturase overexpression cassette and transformation

The *PAD* (Phatr3_J9316) gene was chemically synthesized (Genscript) and codon optimized to remove conflicting restriction sites. The PAD gene was inserted into position 1 in the two‐gene cassette transformation vector pPhOS2 (Hamilton *et al.,* 2014) behind the EF2 promoter (Seo *et al.,* 2015) generating pPhOS2_PAD construct. Construction of the transformation cassette pPhOS2_PAD is described in detail in Data S1. Transformation of *P. tricornutum* Pt4 via biolistic transformation and screening was carried out as described previously (Hamilton *et al.,* 2014).

### Generation of acyl‐ACP Δ9‐desaturase KO lines

A universal KO CRISPR/Cas9 vector was constructed with a dual sgRNA design (Aslan *et al.,* 2015) using dual reporter gene selection system (designed by Mark Youles, personal communication). Type IIS LOOP DNA assembly was used for Level 1 and 2 vector constructions, following the method described in Pollak *et al.* (2019). The design of KO cassettes is explained in detail in Data S1. Transformation of *P*. *tricornutum* Pt4 via biolistic transformation and screening was carried as described previously (Hamilton *et al.,* 2014).

### Cloning of acyl‐ACP Δ9‐desaturase (Phatr3_J9316) into *Synechocystis* expression vector and functional characterization in *Synechocystis*

To generate the Syn_PAD strain, the *Synechocystis* PCC6803 glucose‐tolerant WT (Syn_WT, Himadri Pakrasi, Department of Biology, Washington University, St. Louis, MO, USA) was transformed with the self‐replicative vector pUR (Kim *et al.,* 2016) expressing the acyl‐ACP Δ9‐desaturase gene lacking the 5′ putative signal peptide sequence (as predicted by SignalP online software). Generation of the vector, transformation and functional characterization of the Phatr3_J9316 gene is described in detail in Data S1.

### RNA extraction and quantitative reverse transcription‐PCR

For RNA extraction, 1–1.5 × 10^8^ exponential phase cells were pelleted, flash frozen in liquid nitrogen and stored at −80°C. RNA extraction was carried out using the method described in Rengel *et al*. (2018). cDNA was synthesized and quantitative PCR analysis was carried out as described detail in Data S1.

### Lipid analysis

Whole biomass FAME analysis was carried out as previously reported (Hamilton *et al.,* 2014), using pentadecanoic acid and tricosanoic acid internal standards. Further details are described in Data S1.

Glycerolipids were extracted following a method adapted from Bryant *et al.* (2016), further details are described in Data S1.

For positional analysis, lipids were fractionated by 1D and 2D TLC (described in detail in Data S1), and then purified classes were characterized by preferential loss analysis under low‐energy collision‐induced dissociation as described previously (Abida *et al.,* 2015). After species characterization, quantification of each species was carried out by liquid chromatography‐MS/MS as previously described (Jouhet *et al.,* 2017).

### Transmission electron microscopy

Transmission electron microscopy imaging was carried out by Rothamsted Bioimaging (Harpenden, Herts, UK). Processing of *P. tricornutum* cells for transmission electron microscopy imaging is described in detail in the Data S1.

### Statistics

One‐way analysis of variance was applied to data on specific growth, FA, lipid class and lipid species data. Data were transformed by natural log (quantitative data) or logit (relative %). *Post‐hoc* comparison of the means was carried out using LSD at 5%, 1% and 0.1% level of significance. A two‐tailed Student’s *t*‐test was carried out in cases where only two strains are compared. Microsoft Excel was used for these analyses.

## AUTHOR CONTRIBUTIONS

OS, JAN and VN designed research; RS, JJ and CG performed research; RS, JJ, EM and OS analysed the results RS, OS and JAN wrote the paper.

## CONFLICT OF INTEREST

The authors declare that they have no competing interests.

## Supporting information

**Figure S1.** Schematic map of the expression vector pPhOS2_ *PAD*.
**Figure S2.** Selection of transgenic *P. tricornutum* lines overexpressing an acyl‐ACP Δ9‐desaturase.
**Figure S3.** FA and glycerolipid classes comparison in the WT and overexpressor strains (PAD#16 and PAD#32) during the stationary stage (8 days).
**Figure S4.** Identification of glycerolipids from *P. tricornutum*
**Figure S5.** Generation of cloning cassettes for pRS Ble_DiaCas9_sgRNA2 and KO line analysis.
**Figure S6.** Fatty acids and glycerolipid analysis of the WT and KO strains (∆*pad*#3 and (∆*pad*#7) during the stationary stage of growth phase (8 days).
**Figure S7.** Generation of transgenic SynDes9 strains.
**Figure S8.** Transmission electron micrograph of WT and transgenic strains.
**Figure S9.** Cellular growth of WT and transgenic strains.
**Figure S10.** Proposed pathway for EPA biosynthesis in OE PAD cells.
**Table S1.** The major molecular species for each lipid class in E phase PAD cells as relative (mol %) values.
**Table S2.** The major molecular species for each lipid class in E phase PAD in absolute amounts.
**Table S3.** The major molecular species for each lipid class in S phase PAD cells as relative (mol %) values.
**Table S4.** The major molecular species for each lipid class in PAD cells given in absolute amounts.
**Table S5.** The major molecular species for each lipid class in E phase Δpad cells as relative (mol %) values.
**Table S6.** The major molecular species for each lipid class in E phase Δpad cells given in absolute amounts.
**Table S7.** The major molecular species for each lipid class in S phase Δpad cells as relative (mol %) values.
**Table S8.** The major molecular species for each lipid class in S phase Δpad cells in absolute amounts.
**Table S9.** Fatty acid quantification in major lipid classes of S phase Δpad cells.
**Table S10.**Primers used in this study.
**Data S1.** Supplementary Materials and Methods.

## Data Availability

All relevant data can be found within the manuscript and its supporting materials.
