# Supplementary material for: Plastidial acyl carrier protein Δ9‐desaturase modulates eicosapentaenoic acid biosynthesis and triacylglycerol accumulation in Phaeodactylum tricornutum
Source: Plant J. 2021 May 5;106(5):1247–59. doi: 10.1111/tpj.15231 (PMC8360179; doi:10.1111/tpj.15231)
Supplement: Supplementary file 1 — Figure S1. Schematic map of the expression vector pPhOS2_ PAD. Figure S2. Selection of transgenic P. tricornutum lines overexpressing an acyl‐ACP Δ9‐desaturase. Figure S3. FA and glycerolipid classes comparison in the WT and overexpressor strains (PAD#16 and PAD#32) during the stationary stage (8 days). Figure S4. Identification of glycerolipids from P. tricornutum Figure S5. Generation of cloning cassettes for pRS Ble_DiaCas9_sgRNA2 and KO line analysis. Figure S6. Fatty acids and glycerolipid analysis of the WT and KO strains (∆pad#3 and (∆pad#7) during the stationary stage of growth phase (8 days). Figure S7. Generation of transgenic SynDes9 strains. Figure S8. Transmission electron micrograph of WT and transgenic strains. Figure S9. Cellular growth of WT and transgenic strains. Figure S10. Proposed pathway for EPA biosynthesis in OE PAD cells. Table S1. The major molecular species for each lipid class in E phase PAD cells as relative (mol %) values. Table S2. The major molecular species for each lipid class in E phase PAD in absolute amounts. Table S3. The major molecular species for each lipid class in S phase PAD cells as relative (mol %) values. Table S4. The major molecular species for each lipid class in PAD cells given in absolute amounts. Table S5. The major molecular species for each lipid class in E phase Δpad cells as relative (mol %) values. Table S6. The major molecular species for each lipid class in E phase Δpad cells given in absolute amounts. Table S7. The major molecular species for each lipid class in S phase Δpad cells as relative (mol %) values. Table S8. The major molecular species for each lipid class in S phase Δpad cells in absolute amounts. Table S9. Fatty acid quantification in major lipid classes of S phase Δpad cells. Table S10.Primers used in this study. Data S1. Supplementary Materials and Methods. [file TPJ-106-1247-s001.docx]

**Supporting Information**

**Supplementary Figures**

A

MLSTKLFWTSSVLASSAVLA**F**PTSPATRTPRSTSILKVAVDPTTVTKKEYEDICGVSFDADTLEDRLKATNYLYPKHVEVIDDIAPIAGAMVDEILLETGENAWQPQDYLPDLSQDNWHDSIKEVRAMAKEIPDELLVVLIGDMVTEEALPTYQTLLNTFEGCDDPTGTSESPWARWSRGWTSEENRHGDLLNKYLYLGGRCDMRNIEVTIQHLITNGFNPQARKDPYRGFVYTSFQERATKISHGNVGKLARTYGEKNLNKICAKIAGDEGRHEKAYQIFSEEILKRDPDGLIHVFGDMMRGQIVMPAEQMTDGKDPDLYDNFSMVAQKTGVYTALDYAEIIDHLVKRWDLEHLEGLSPAAEKEREYLCRL PERYRKLATRSMNKKKKVTEDEDPLKSFGWIYGRMA

B

**Fig. S1.** Schematic map of the expression vector pPhOS2_ *PAD*. (*A*). Amino acid sequence of the Phat3_J9316 gene. The phenylalanine (F) conserved in the “ASAFAP” motif of diatoms plastid bipartite targeting signal is highlighted in red. (*B*) The synthetic Phat3_J9316 gene was fused to the *P. tricornutum* FcpA terminator and cloned into position 1 under control of the native EF2 promoter (2). Position 3 is occupied by a zeocin resistance gene (*Ble)* flanked by FcpB promoter and FcpA terminator. MCS- Multicloning site of the Position 2.


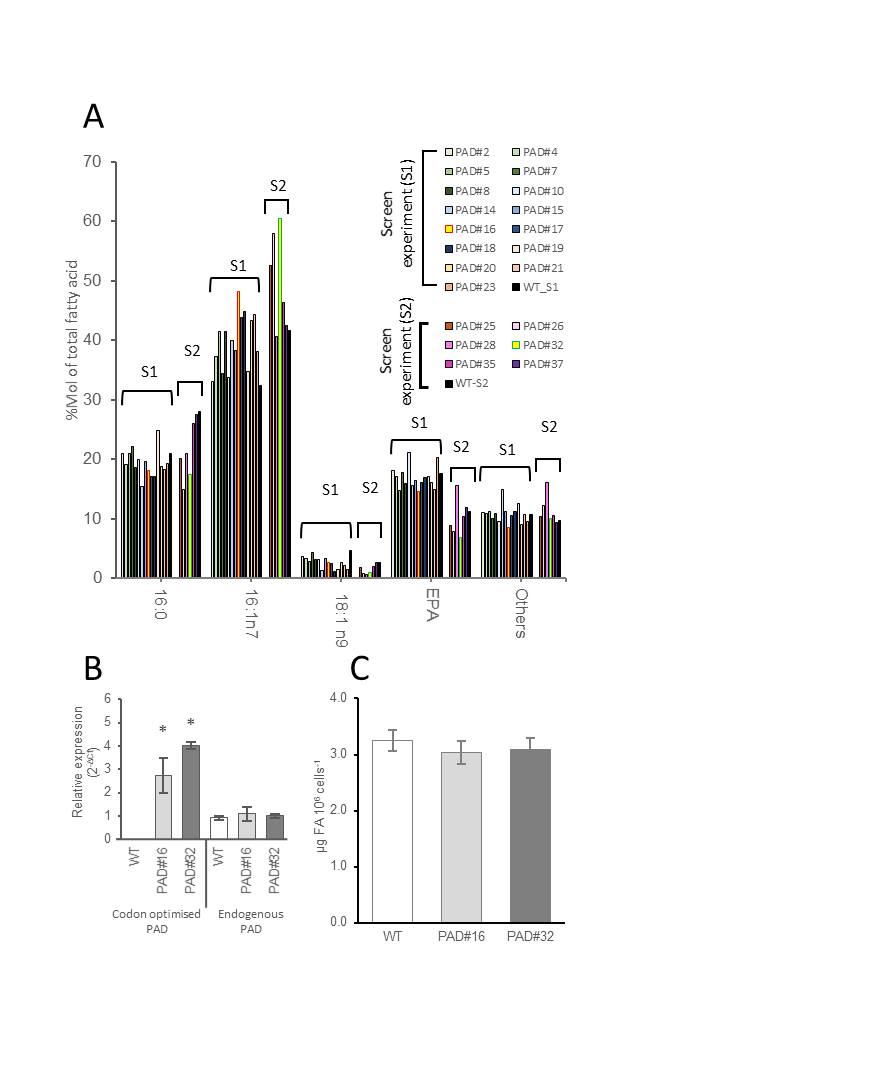


**Fig. S2.** Selection of transgenic *P. tricornutum* lines overexpressing an acyl-ACP Δ9-desaturase. (A) Fatty acid composition (Mol %) of selected independent lines overexpressing Phat3_J9316 gene during late exponential (E) phase. S1 and S2 relate to two independent screening experiments with different harvesting times. (*B*) Transcript abundance of synthetic and endogenous Phatr3_J9316 genes. Expression is reported relative to the reference gene Aureochrome (Phatr3_J8113). *(C*) Total fatty acid content expressed in µg FA 10^6^ cells^-1^ WT and transgenic Δ9-desaturase overexpression lines during exponential phase. Values presented are the average of three biological replicates, error bars represent SE. Asterisks indicate significant difference relative to WT (p<0.05, LSD).


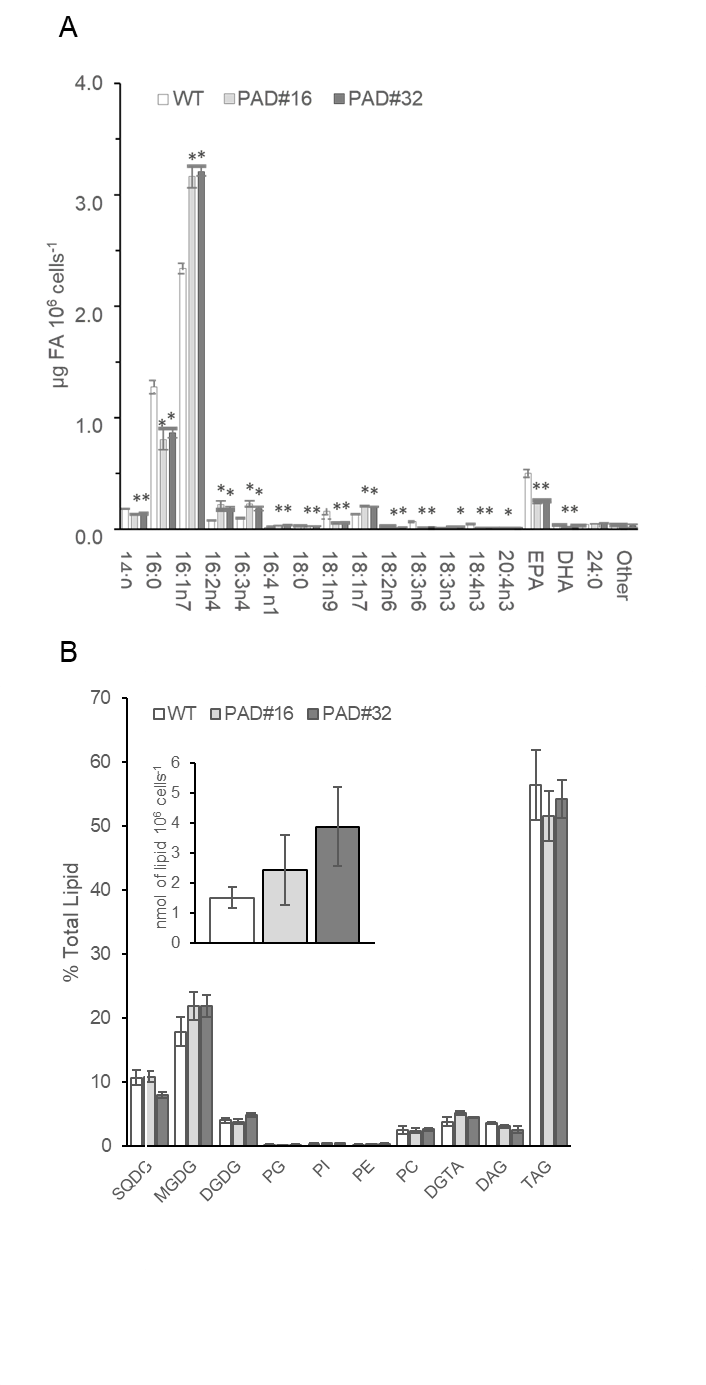


**Fig. S3.**  FA and glycerolipid classes comparison in the WT and overexpressor strains (PAD#16 and PAD#32) during the stationary stage (8 days). (*A*) FA content. Values expressed in µg FA 10^6^ cells^-1^. (*B*) Glycerolipid classes. Values expressed in % of total lipids. Insert: Quantitative analysis of total glycerolipids (nmol of lipid 10^6^ cells^-1^). Growth stage was determined by analysing cell density. Values presented are the average of three biological replicates, error bars represent SE. Asterisks indicate significant difference relative to WT (p<0.05, LSD).


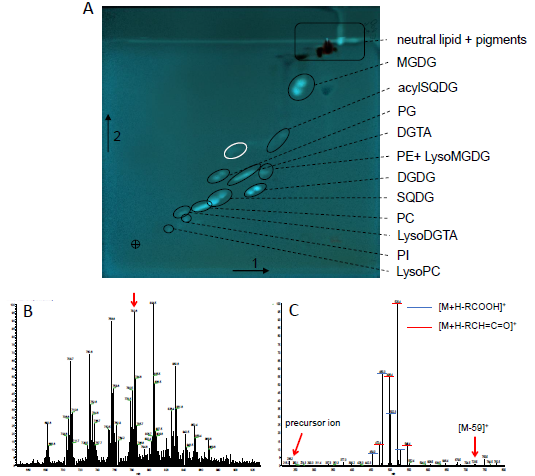


**Fig. S4.** Identification of glycerolipids from *P. tricornutum*. (*A*) Two-dimensional TLC separation. Lipids from PT4 cells grown for 4 days to mid-log phase (1.9 x 10^-6^ cells /ml) were extracted and resolved as described in “Methods”. The cross indicates the initial deposit. The spot circled in white is an unidentified non-glycerolipid compound. (*B*) Full scan LC-MS analysis of the DGTA spot isolated by two-dimensional thin layer chromatography in positive mode. (*C*) MS2 fragmentation of the 782 m/z ion. Arrow heads indicate fragments corresponding to the DGTA signature. Precursor m/z 236 corresponds to the polar head mass of either DGTA or DGTS; the fragment 723 results from a neutral loss of 59 from m/z 756 that is characteristic of DGTA and DGTS; absence of a m/z 695 corresponding to a neutral loss of 87 characteristic to DGTS (13).


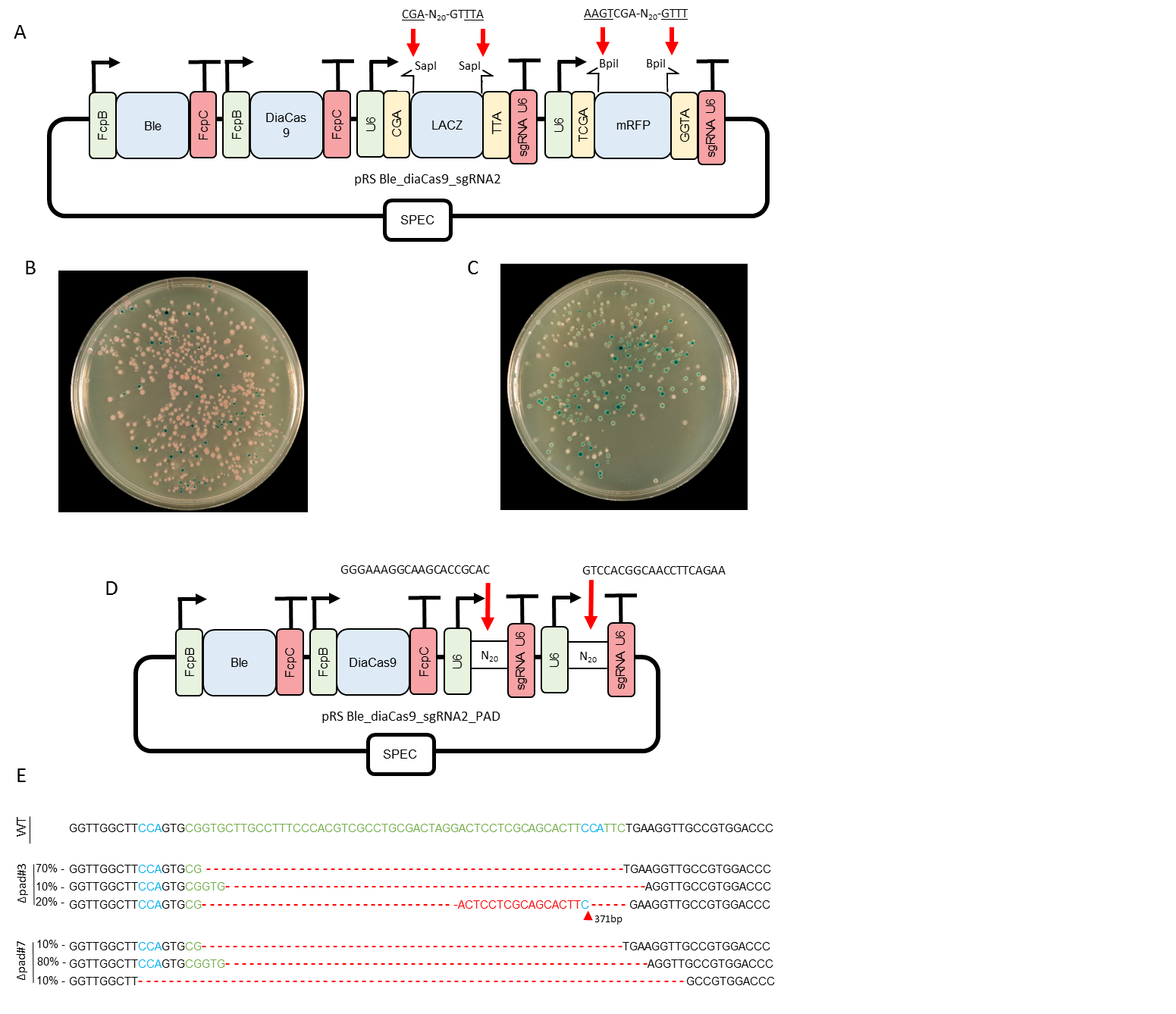


**Fig. S5**. Generation of cloning cassettes for pRS Ble_DiaCas9_sgRNA2 and KO line analysis. (*A*) pRS_diaCas9_sgRNA2 vector map, red arrows indicate guide insertion sites and unique overhangs. (*B*) Successful insertion of the position 1 sgRNA resulted in red colonies. (*C*) Successful insertion of both sgRNAs resulted in white colonies, negative colonies are blue. (*D*) The final pRS_diaCas9_PAD vector contains both guides with identical fusion to upstream U6 promoter and downstream sgRNA. (*E*) Deletion sequencing. Sequences of PCR product of the Phat3_J9316 gene in Δ*pad3* and Δ*pad*7 colonies. The red arrow indicates the site of DNA insertion.


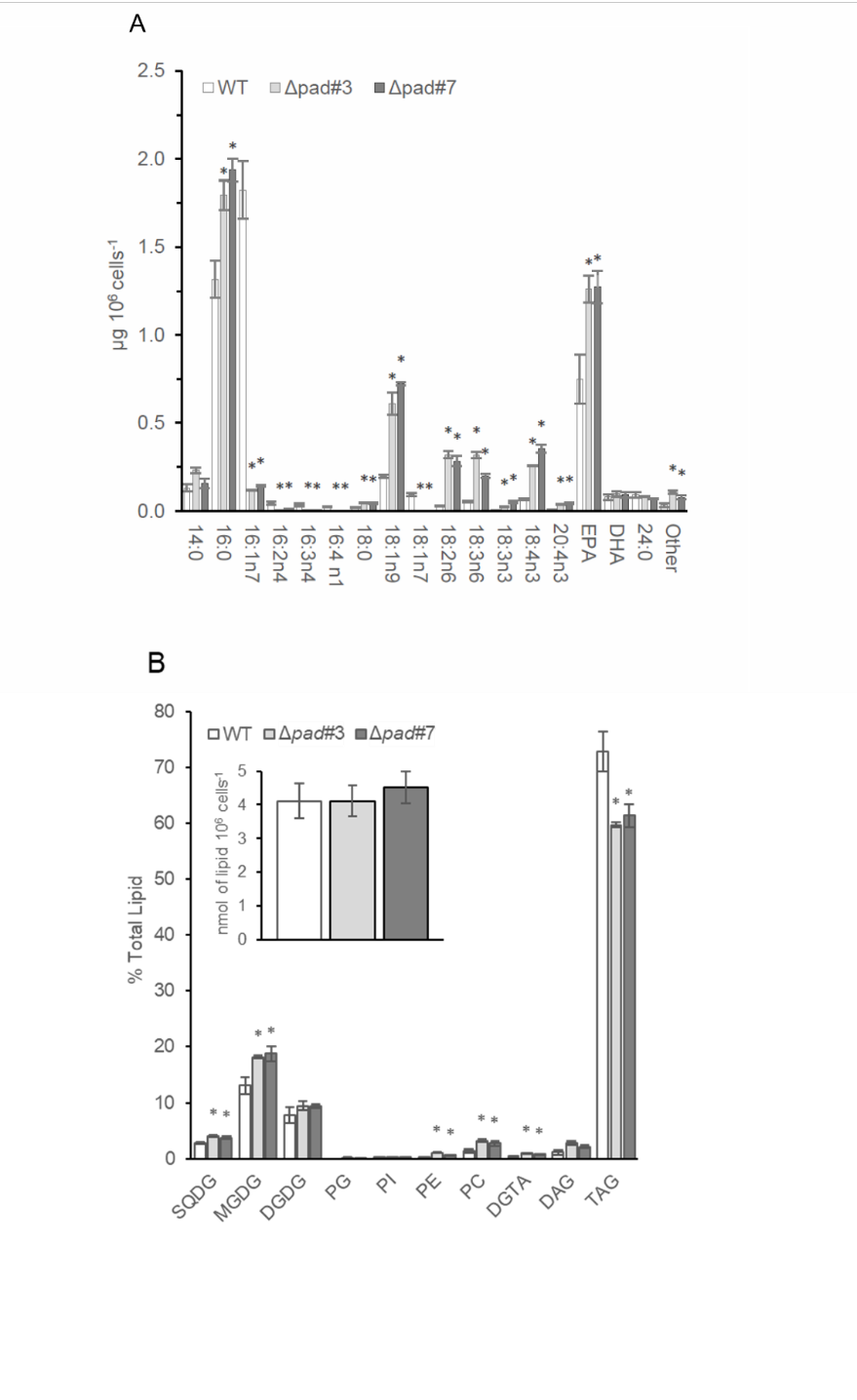


**Fig. S6**. Fatty acids and glycerolipid analysis of the WT and KO strains (∆*pad*#3 and (∆*pad*#7) during the stationary stage of growth phase (8 days). (*A*) FA content. Values expressed in µg FA 10^6^ cells^-1^. (*B*) Glycerolipid classes. Values expressed in % of total lipids. Insert: Quantitative analysis of total glycerolipids (nmol of lipid 10^6^ cells^-1^). Growth stage was determined by analysing cell density. Values presented are the average of three biological replicates, error bars represent SE. Asterisks indicate significant difference relative to WT (p<0.05, LSD).


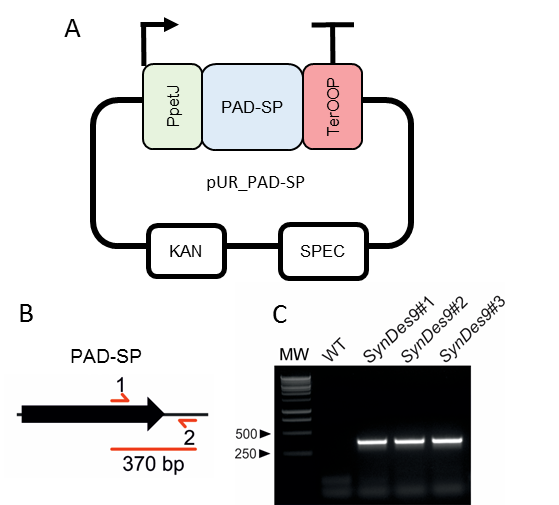


**Fig S7. Generation of transgenic SynDes9 strains.** (*A*) Map of the plasmid pUR_PAD-SP. pUR9D-SP was generated by cloning the Phat3_J9316 gene lacking the predicted signal peptide downstream the P_petJ_ promoter in the replicative pUR vector (14). (*B*) The scheme shows the annealing position of the primers used to screen for positives transformants and the expected amplicon size. (*C*) Colony PCR (Table S10) shows three independently isolated positive colonies (SynDes9#1, 2, 3).


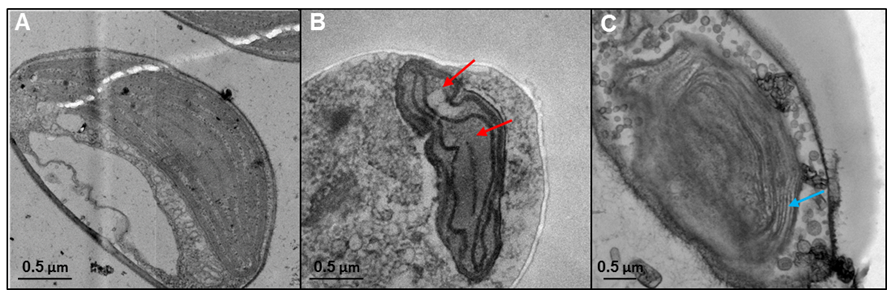


**Fig. S8.** Transmission electron micrograph of WT and transgenic strains. (*A*) WT chloroplast. (*B*) The overexpressor PAD chloroplast. (*C*) The KO chloroplast. Red and blue arrows represent thylakoid stacks.


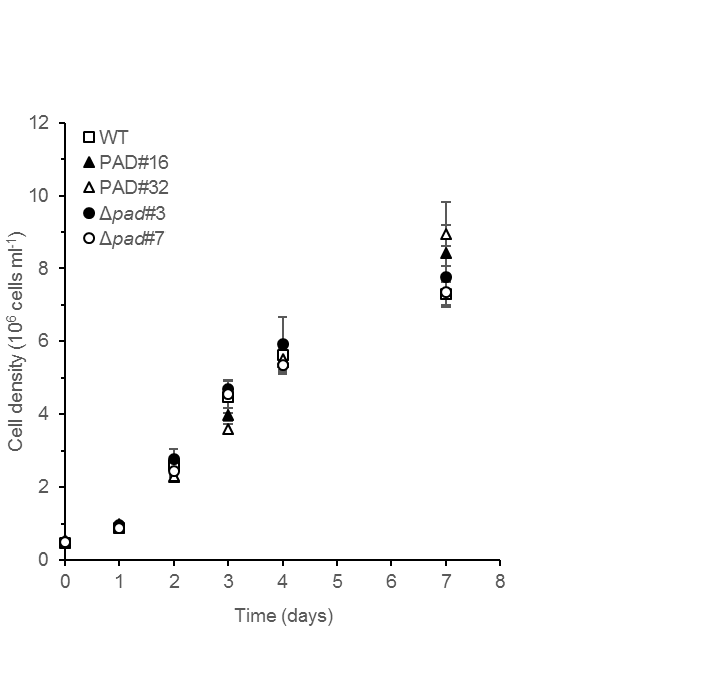


**Fig. S9.** Cellular growth of WT and transgenic strains. Values presented are the average of three biological replicates, error bars represent SE. No significant difference was found in specific growth rates between cell strains (ANOVA, p<0.05). Cells grown under 100 umol m^-2^ s^-1^


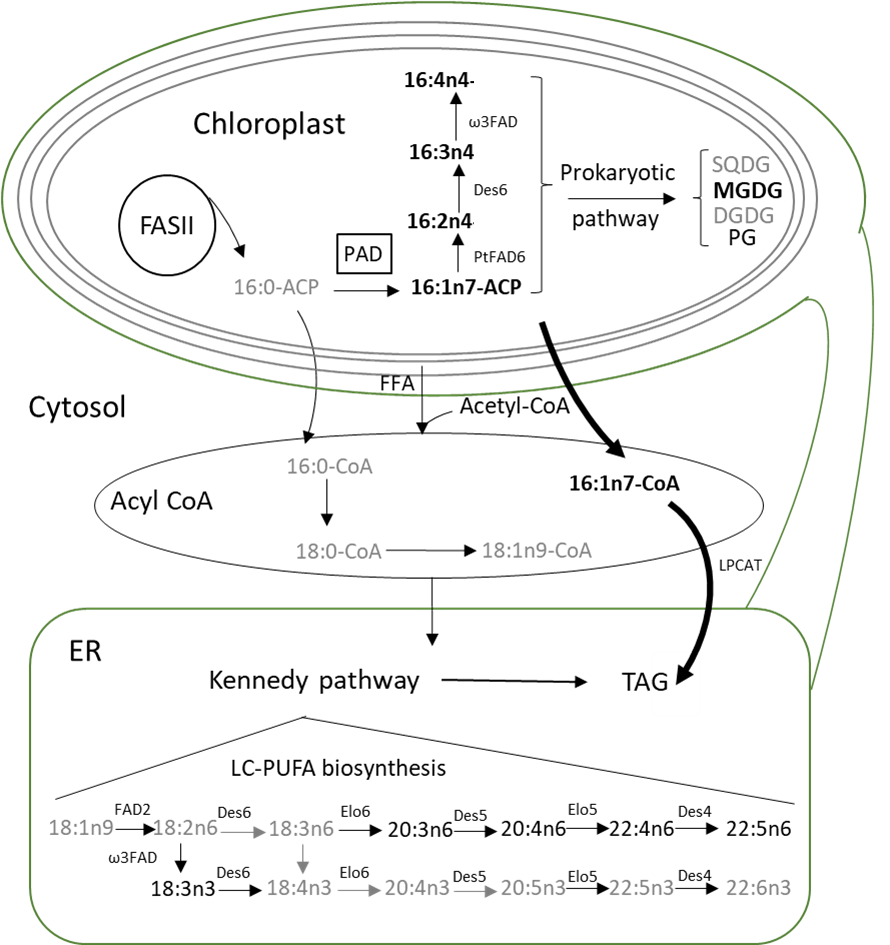


**Fig. S10. Proposed pathway for EPA** **biosynthesis in OE PAD cells.** C16 FA generated in chloroplast are exported for ER editing or retained in the stroma for the “prokaryotic” pathway. Exported C16 are used for the production of TAG *via* the Kennedy pathway and for LC-PUFA biosynthesis. Bold text indicates increased pool size of metabolite or activity of enzyme, grey text indicates reduced pool size or activity of enzyme.

Supplementary Tables

Table S1. The major molecular species for each lipid class in E phase PAD cells as relative (mol %) values. (*A*) Plastidial lipids. (*B*) Extraplastidial lipids.

A

B

Table S2. The major molecular species for each lipid class in E phase PAD cells are given in absolute amounts (pmol 10^6^cells ^-1^). (*A*) Plastidial lipids. (*B*) Extraplastidial lipids.

A

B

Table S3 The major molecular species for each lipid class in S phase PAD cells as relative (mol %) values. (*A*) Plastidial lipids. (*B*) Extraplastidial lipids.

A

B

A

Table S4. The major molecular species for each lipid class in PAD cells are given in absolute amounts (pmol 10^6^cells ^-1^), S phase. (*A*) Plastidial lipids. (*B*) Extraplastidial lipids.

A

B

Table S5. The major molecular species for each lipid class in E phase Δpad cells as relative (mol %) values. (*A*) Plastidial lipids. (*B*) Extraplastidial lipids.

A

B

Table S6. The major molecular species for each lipid class in E phase Δpad cells are given in absolute amounts (pmol 10^6^cells ^-1^). (*A*) Plastidial lipids. (*B*) Extraplastidial lipids.

A

B

Table S7. The major molecular species for each lipid class in S phase Δpad cells as relative (mol %) values. (*A*) Plastidial lipids. (*B*) Extraplastidial lipids.

A

B

Table S8. The major molecular species for each lipid class in S phase Δpad cells in absolute amounts. (*A*) Plastidial lipids. (*B*) Extraplastidial lipids.

A

B

Table S9. Fatty acid quantification in major lipid classes of S phase Δpad cells. (*A*) Plastidial lipids. (*B*) Extraplastidial lipids.

A

B

Table S10. Primers used in this study.

**Supplementary Materials and Methods**

**Construction of PAD overexpression cassette.** The transformation cassette pPhOS2_PAD was constructed by modifying the pPhOS2 vector containing a zeocin resistance gene (*Ble)* flanked by FcpB promoter and FcpA terminator, as previously described (1). The endogenous elongation factor 2 (EF2) promoter was chosen to drive expression due to evidence of improved stability throughout light and dark cycles and higher expression under both nitrogen replete- and deplete conditions relative to FcpA (2). The promoter was cloned from *P. tricornutum* gDNA with the addition of *PstI* and *EcoRI* restriction sites 5’ upstream and 3’ terminal flank, respectively (primers shown in Table S10) and an optimised CCACC Kozak sequence. The EF2 promoter and *PAD* gene were subsequently ligated into position 1 of the pPhos2 vector together using the restriction enzymes Pst1 and EcoRV, replacing the FcpA promoter but retaining the FcpA terminator (Fig. S1).

**Generation of PAD KO Cassettes.** For the construction of universal knock-out CRISPR/Cas9 vector first the level 0 vectors were constructed by cloning a codon optimised for expression in *P.tricornutum* Cas9 gene, endogenous U6 promoter, sgRNA module and U6 termination signal from the pks diaCas9_sgRNA vector (a gift from Per Winge, Addgene plasmid # 74923, (3) into a universal acceptor plasmid (pAGM9121, a gift from Sylvestre Marillonnet, Addgene plasmid # 51833). Primers used for this cloning are detailed in Table S10. Two versions of the U6 promoter and sgRNA scaffold were created with unique (3’ and 5’, respectively) restriction site overhangs to enable site specific ligations of the guide RNA. Level 0 vectors containing domesticated FcpB promoter, FcpC terminator and Ble (conferring zeocin resistance) were kindly provided by Dr. Katrin Geisler. Reporter genes mRFP and LACZ were cloned from pICSL00217A vector (kindly provided by Dr. Mark Youles) with the addition of *BpiI* (outward facing) and *BsaI* (inward facing) restriction sites.

For construction of Level 1 vectors for pRS Ble_DiaCas9_sgRNA2, the Ble gene and diaCas9 were assembled with FcpB promoter and FcpC terminator into pOdd_1 and pOdd_2 Loop destination vectors (a gift from Dr Pollack (4). Components of the sgRNA transcriptional unit were ligated with the reporter genes PCR products, to produce two unique sgRNA units with internal reporter genes LACZ and mRFP, using pOdd_3 and pOdd_4 destination vectors.

For the level 2 vector, the pEven_1 vector (a gift from Dr. Pollack) was first domesticated to replace BpiI with EcoRV using Q5 Site-Directed Mutagenesis (NEB), creating a modified vector pEven_1m. The Level 1 Ble, diCas9, sgRNA (LACZ) and sgRNA (mRFP) transcriptional units were then inserted into pEven_1m. Following ligation problems, the LACZ with outward facing BpiI sites was replaced with LACZ with outward facing SapI (Fig. S5*A*). The guide RNAs were constructed from annealed ssDNA oligonucleotides (Table S10). The oligonucleotides were mixed at 50µM in an annealing buffer (10 mM TRIS pH 8, 50 mM NaCl, 1mM EDTA), heated to 95°C for 5 minutes then left to cool slowly to room temperature. The resulting dsDNA guides were ligated into the pRS Ble_diaCas9_sgRNA2 vector in a two-step digestion-ligation reaction using Type IIS enzymes BpiI and SapI, to directly insert guide RNAs (100:1 molar ratio). The first digestion with SapI followed by ligation with guide 1 resulted in replacement of the LACZ gene leaving cells expressing the mRFP gene, within sgRNA2, alone (red colonies) (Fig. S5*B*). The second digestion with BpiI and ligation with the second guide, replacing the mRFP gene, left positive colonies white Fig. S5*C*. This protocol led to the generation of the pRS Ble_diaCas9_sgRNA2_PAD vector (Fig. S5*D*).

*P. tricornutum* colonies, transformed with the pRS Ble_diaCas9_sgRNA2_PAD were screened via PCR for the diaCas9 gene and a band shift change of the PAD gene using primers outlined in Table S10. PCR product (Fig. 2*B* and Table S10) was cloned into a Zero Blunt™ TOPO™ vector and transformed into *E. coli.* Ten resistant colonies from each PCR product were sequenced to determine sequence diversity. Sequencing of the PCR product revealed a mosaicism in within each KO line (Fig. S5*E*). Δ*pad#3* had a mixture of deletions between position 51bp and 107bp (70% of cloned PCR product), 54bp and 109 (10%), and an insert of 23 bp of the PAD gene (79-102 bp) and 371bp (red arrowhead) from the vector backbone between positions 51 and 108 (20%). Δ*pad#7* has a similar diversity cutting between positions 44 and 115 (10%), 51 and 107 (10%), and 54 and 109 (80%). No WT sequence within the KO target was detected.

**Generation of Synechocystis transgenic lines and functional characterisation of PAD.**

The *PAD* gene was amplified by PCR from the pPhos2_PAD vector (primers detailed in Table S10), digested by *NdeI* and *BamHI* and cloned downstream from the inducible P_petJ_ promoter of pUR, generating the vector pUR_PAD-SP (Fig. S7). The WT strain was transformed with pUR_PAD-SP by triparental mating conjugation as described previously (5). Transformants were selected on kanamycin- and spectinomycin-containing BG-11 agar plates and the presence of the gene verified by PCR.

*Syn_*WT (kindly provided by Himadri Pakrasi, Department of Biology, Washington University, St Louis, MO, USA) and a mutant strain (Syn_PAD) were maintained under continuous light (50 µmol photons m^-2^s^-1^) at 28°C in BG-11 medium (6). Copper-free BG-11 (hereafter BG-11woCu) was prepared omitting CuSO_4_ from the trace metal mix. Cells were washed in BG-11woCu medium two times before starting the cultivation in the absence of copper, in order to induce the P_petJ_ promoter (7). Syn_WT and Syn_PAD strains were grown in BG-11woCu until mid-exponential phase (0.6 OD_750_), diluted to 0.05 OD_750_ in BG-11woCu medium and aliquoted in 24-wells plates, 2mL for each well. C16:0, C18:0 or C16:1 fatty acids were exogenously supplied at 75 μm final concentration (8). The plates were incubated on a shaker for 4 days to reach 0.4 OD_750_ before being harvested for FAME analysis.

**Lipid Analysis**. For whole biomass FAME analysis, a 5 ml aliquot of cell culture in exponential (3 days growth) or stationary phase (8 days growth) was pelleted and subsequently dried under N_2_. After desiccation, samples were derivatised with the addition of 1 ml of methylation mix containing Methanol, Toluene, and H_2_SO_4_ (35:14:1 ratio) and 6.25 µg ml^-1^ of pentadecanoic acid (C15:0) and tricosanoic acid (C23:0) internal standards (9). The mix was incubated at 85°C for 90 minutes. Following methylation 1 ml 1% w/v NaCl and 1 ml Heptane was added to each sample. Samples were centrifuged for 5 minutes to aid phase separation. A 300 µl aliquot of the heptane fraction was dried under N_2_ and resuspended in heptane with 0.01% w/v BHT. Methyl ester derivatives of the total fatty acids (FAMEs) were separated and quantified by GC-FID (Agilent 7890A) using an Agilent DB-23 column (30 m, 0.25 mm, 0.25 µm). Peaks were identified by comparison of retention times with Supleco 37 FAME Mix (Sigma) and methylated Qual Mix Menhaden fish oil (Larodan). Internal standards were used to quantify FAMEs.

Glycerolipids for extracted cells grown in 50 ml of culture in exponential (3 days growth) or stationary phase (8 days growth). Separately, a larger sample from 200 ml of culture were harvested in exponential (4 days growth) for structural analysis of lipid classes. Cells were flash frozen in liquid N_2_, stored at -80°C, then lyophilized. Lipids were extracted following method adapted from (10). Cell pellets were boiled in 4ml ethanol for 10 minutes to denature lipases. The mixture was added to 10 ml chloroform/methanol (4:1), flushed with N_2_, then stirred for 1 hour at room temperature. The mixture was filtered through glass wool to remove debris. The glass wool was then rinsed with 3 ml chloroform/methanol (2:1). To induce phase separation 5ml 1% NaCl was added to the filtered mixture. The phases were vortexed for 10 seconds, then separated by centrifugation for 5 minutes. The bottom polar phase was extracted and dried under N_2_, resuspended in 500 µl chloroform and stored at -80°C. A 1/100 aliquot was taken for FA quantification using the method outlined in above.

To determine positional distribution of fatty acid species in each lipid class, total lipids were first separated by thin-layer chromatography (TLC) using glass-backed silica gel plates (Merck) as described in (11). Neutral lipid were resolved in one-direction using hexane:diethylether:acetic acid (70:30:1, v/v). Membrane glycolipids were separated in two directions with chloroform:methanol:water (65:25:4, v/v), then acetone: methanol:acetic acid:water (50:20:10:10:5, v/v) after drying overnight in an argon chamber. Silica plates were sprayed with 0.2% w/v 8-anilino-1-naphthanlenesulfonic acid in method then visualised under UV light. Lipid classes were identified, scrapped off the plate and extracted with the sequential addition and agitation of 1.35 chloroform/methanol (1:2), 0.45 ml chloroform, and 0.8 ml H_2_0. After centrifugation, the chloroform layer was recovered and dried under argon. Lipids were dissolved in 10 mM ammonium acetate in methanol, and analysed by direct-infusion (electrospray ionisation-MS) into a ion trap mass spectrometer (LTQ-XL, Thermo Fisher Scientific). Lipid classes were confirmed by MS2 analysis through neutral loss analysis or their precursor ion (11). Positional analysis was carried out as described in Abida et al., 2015, with preferential loss analysis under low-energy collision-induced dissociation (11). Quantification of lipid species was achieved by LC-MS2 analysis, normalised with a number of quality control (previously characterised by TLC and GC-FID), as detailed in (12).

**Transmission Electron Microscopy.** For transmission electron microscopy, cells were pelleted (Algae were centrifuged at 3000rpm for 5 minutes to pellet. The supernatant was removed and the pellet resuspended in 2% low melting point agarose at 37C. The algae agarose mixture was pipetted into 6mm planchettes (Leica) and high pressure frozen using a Leica Microsystems EM HM 100. Samples were stored in liquid nitrogen before freeze substitution using acetone and osmium tetroxide in a Leica Microsystems EM AFS. Following freeze substitution samples were stored at -20C then 4C for 24 hours each. The samples were infiltrated with a dry acetone:Spurr resin series and polymerised at 60C. 50nm ultrathin sections were cut using a Leica Microsystems UC7 ultra microtome and collected on copper grids (Agar) coated with formvar and carbon. The sections were post stained with uranyless (TAAB) and lead citrate (TAAB) to increase constrast. Images were obtained using a JEOL 2011 transmission electron microscope at 200kV and a GATAN Ultrascan CCD camera.

1. M. L. Hamilton, R. P. Haslam, J. A. Napier, O. Sayanova, Metabolic engineering of Phaeodactylum tricornutum for the enhanced accumulation of omega-3 long chain polyunsaturated fatty acids. *Metabolic Engineering* **22**, 3-9 (2014).

2. S. Seo, H. Jeon, S. Hwang, E. Jin, K. S. Chang, Development of a new constitutive expression system for the transformation of the diatom Phaeodactylum tricornutum. *Algal Research-Biomass Biofuels and Bioproducts* **11**, 50-54 (2015).

3. M. Nymark, A. K. Sharma, T. Sparstad, A. M. Bones, P. Winge, A CRISPR/Cas9 system adapted for gene editing in marine algae. *Scientific Reports* **6** (2016).

4. B. Pollak *et al.*, Loop assembly: a simple and open system for recursive fabrication of DNA circuits. *New Phytologist* **222**, 628-640 (2019).

5. V. V. Zinchenko, I. V. Piven, V. A. Melnik, S. V. Shestakov, Vectors for the complementation analysis of cyanobacterial mutants. *Russian Journal of Genetics* **35**, 228-232 (1999).

6. R. Rippka, J. Deruelles, J. B. Waterbury, M. Herdman, R. Y. Stanier, GENERIC ASSIGNMENTS, STRAIN HISTORIES AND PROPERTIES OF PURE CULTURES OF CYANOBACTERIA. *Journal of General Microbiology* **111**, 1-61 (1979).

7. E. Kuchmina, T. Wallner, S. Kryazhov, V. V. Zinchenko, A. Wilde, An expression system for regulated protein production in Synechocystis sp PCC 6803 and its application for construction of a conditional knockout of the ferrochelatase enzyme. *Journal of Biotechnology* **162**, 75-80 (2012).

8. F. Domergue *et al.*, New insight into Phaeodactylum tricornutum fatty acid metabolism. Cloning and functional characterization of plastidial and microsomal Delta 12-fatty acid desaturases. *Plant Physiology* **131**, 1648-1660 (2003).

9. R. Garces, M. Mancha, ONE-STEP LIPID EXTRACTION AND FATTY-ACID METHYL-ESTERS PREPARATION FROM FRESH PLANT-TISSUES. *Analytical Biochemistry* **211**, 139-143 (1993).

10. D. Simionato *et al.*, The Response of Nannochloropsis gaditana to Nitrogen Starvation Includes De Novo Biosynthesis of Triacylglycerols, a Decrease of Chloroplast Galactolipids, and Reorganization of the Photosynthetic Apparatus. *Eukaryotic Cell* **12**, 665-676 (2013).

11. H. Abida *et al.*, Membrane Glycerolipid Remodeling Triggered by Nitrogen and Phosphorus Starvation in Phaeodactylum tricornutum. *Plant Physiology* **167**, 118-136 (2015).

12. J. Jouhet *et al.*, LC-MS/MS versus TLC plus GC methods: Consistency of glycerolipid and fatty acid profiles in microalgae and higher plant cells and effect of a nitrogen starvation. *Plos One* **12** (2017).

13. I. Armada *et al.*, Differences in betaine lipids and fatty acids between Pseudoisochrysis paradoxa VLP and Diacronema vlkianum VLP isolates (Haptophyta). *Phytochemistry* **95**, 224-233 (2013).

14. A. Wiegard *et al.*, Biochemical analysis of three putative KaiC clock proteins from Synechocystis sp PCC 6803 suggests their functional divergence. *Microbiology-Sgm* **159**, 948-958 (2013).
